# Associations of fetal and infant growth patterns with behavior and cognitive outcomes in early adolescence

**DOI:** 10.1002/jcv2.12278

**Published:** 2024-10-23

**Authors:** Romy Gonçalves, Romy Gaillard, Kelly K. Ferguson, Sara Sammallahti, Manon H. Hillegers, Eric A. P. Steegers, Hanan El Marroun, Vincent W. V. Jaddoe

**Affiliations:** ^1^ The Generation R Study Group Erasmus University Medical Center Rotterdam the Netherlands; ^2^ Department of Pediatrics Sophia’s Children’s Hospital Erasmus University Medical Center Rotterdam the Netherlands; ^3^ Department of Health and Human Services Epidemiology Branch National Institute of Environmental Health Sciences National Institutes of Health Durham NC USA; ^4^ Department of Child and Adolescent Psychiatry/Psychology Erasmus University Medical Center Rotterdam the Netherlands; ^5^ Department of Social and Behavioral Sciences Harvard School of Public Health Boston MA USA; ^6^ Department of Obstetrics and Gynecology University of Helsinki and Helsinki University Hospital Helsinki Finland; ^7^ Department of Obstetrics and Gynecology Sophia’s Children’s Hospital Erasmus University Medical Center Rotterdam the Netherlands; ^8^ Department of Psychology Education and Child Studies Erasmus School of Social and Behavioral Sciences Erasmus University Medical Center Rotterdam the Netherlands

**Keywords:** attention‐deficit hyperactivity disorder, autism spectrum disorder, behavior, cognitive development, fetal growth, infant growth, IQ

## Abstract

**Background:**

Fetal life and infancy might be critical periods for brain development leading to increased risks of neurocognitive disorders and psychopathology later in life. We examined the associations of fetal and infant weight growth patterns and birth characteristics with behavior and cognitive outcomes at the age of 13 years.

**Methods:**

Population‐based prospective cohort study from fetal life until adolescence. Pregnant women with a delivery date between April 2002 and January 2006 were eligible. Follow‐up measurements were available for 4716 children. Fetal weight was estimated in the second and third trimesters of pregnancy by ultrasonography. Infant weight was measured at birth and at 6, 12, and 24 months. Fetal and infant weight acceleration or deceleration were defined as a change in SD greater than 0.67 between time points. Total, internalizing and externalizing problems and attention‐deficit hyperactivity disorder (ADHD) symptoms were measured using Child Behavior Checklist (CBCL/6–18), autistic traits by the Social Responsiveness Scale (SRS) and intelligence quotient (IQ) by the Wechsler Intelligence Scale for Children‐Fifth Edition (WISC‐V).

**Results:**

One week longer gestational age at birth was associated with a −0.03 SDS (95% Confidence Interval (CI): −0.04, −0.01) lower total behavior problems score, a −0.02 SDS (95% CI: −0.04, −0.01) lower ADHD symptoms score. Also an increase in birth weight of 500 g was associated with a lower odds of having high externalizing problems (OR 0.92 (95% CI: 0.86, 0.98) and of having a low IQ score (OR 0.79 (95% CI: 0.71, 0.88). Compared to children with normal fetal and infant growth, those with accelerated fetal and infant growth had a 0.27 SDS higher IQ (95% Confidence Interval 0.11, 0.44).

**Conclusions:**

Both fetal and infant weight development are associated with behavioral and cognitive outcomes in early adolescence. Follow‐up studies are needed to assess whether these associations link to later life mental health outcomes.

**Key points**

**What’s known?**

- **In early life rapid development of the central nervous system takes place. Fetal and early life weight growth might be important for neurocognitive development.**

**What’s new?**

- **In this large population‐based prospective study we observed that both birth characteristics and fetal and infant growth patterns are associated with behavior and cognitive outcomes in early adolescence. compared to children with normal fetal and infant growth, those with accelerated fetal and infant growth had the highest iq.**

**What’s relevant?**

- **Fetal and early life growth patterns that effect neurocognitive development have not yet been identified. This study gives an insight into the possible window of opportunity in early adolescent behavioral and cognitive development provided by fetal and early life weight growth.**

## INTRODUCTION

Preterm birth and low birth weight are associated with increased risks of impaired cognitive development, psychopathology and intellectual disabilities (Anderson et al., 2021; Hack et al., 2005; Johnson & Marlow, 2011). Low birth weight is also associated with poor performance on tests for neurocognitive function, attention‐deficit hyperactivity disorder (ADHD) and autism spectrum disorder (ASD) among children, adolescents and adult (Sacchi et al., 2020; Song et al., 2022; Talmi et al., 2020). These associations are stronger among the extremes of birth characteristics but present across the normal range of gestational age and weight at birth (Anderson et al., 2021; Cortese et al., 2021; Pettersson et al., 2019; van Mil et al., 2015). Gestational age and weight at birth may not be causal factors per se leading to behavior and cognition later in life, but are merely markers of a continuous growth process that started before birth. A recent study from our group reported that among small for gestational age (SGA) children, those consistently small from mid‐pregnancy onwards had the lowest IQ and most ADHD symptoms (Ferguson et al., 2021). Another, large individual participant data meta‐analysis in very preterm and low birthweight children showed much higher risks of meeting the criteria for ADHD, ASD, Anxiety disorder and Mood disorder (Anderson et al., 2021). Also, a recent study in Belarus among 11,000 children reported that faster weight growth from birth to 5 years was positively associated with intelligence quotient (IQ) at 6 years old (Yang et al., 2011). Fetal life and early childhood are characterized by rapid development of the central nervous system (Konkel, 2018). Both early life weight and head circumference are related to cognitive skills in childhood (Bach et al., 2020; Geraedts et al., 2011). We have previously reported that increased weight gain during the fetal period and in infancy is associated with larger brain volumes on MRI scans at 10 years of age (Silva et al., 2021). Prospective studies on the associations of different fetal and infant growth characteristics with neurocognitive and psychopathology outcomes in childhood may contribute to identification of specific critical periods and windows of opportunity in fetal and infant growth. We hypothesize that birth characteristics and different fetal and infant growth patterns are associated with behavior and cognitive outcomes in early adolescence.

In this population‐based prospective cohort study among 4716 children, we examined the associations of fetal and infant weight growth patterns and birth characteristics with behavior and cognitive outcomes at the age of 13 years. Main outcomes included parent‐reported scores for total behavior problems, internalizing behavior problems reflecting anxiety, depressive and somatic symptoms and externalizing behavior problems reflecting rule breaking and aggressive behavior, ADHD symptoms, ASD traits and observed IQ. We were specifically interested in identification of specific fetal and infant growth patterns.

## METHODS

### Study population

This study was embedded in the Generation R Study, a population‐based prospective cohort study from early fetal life onwards (Kooijman et al., 2016). Pregnant women with a delivery date between April 2002 and January 2006, living in Rotterdam, the Netherlands, were eligible. Details on response and follow‐up were described previously (Kooijman et al., 2016). We had information on fetal or infant growth in 8624 singleton births. Analyses were restricted to a subgroup of (*n* = 4716) children for whom we had follow‐up information at 13 years (follow‐up rate 54.7%). A flowchart is given in the Supplemental Figure **S1**.

### Ethical considerations

Written informed consent was provided by all parents and children. The Medical Ethics Committee of Erasmus Medical Center approved the study (MEC‐2012‐165). This study followed the Strengthening the Reporting of Observational Studies in Epidemiology (STROBE) reporting guideline (Vandenbroucke et al., 2007).

### Fetal and infant growth measures

As previously described, fetal ultrasound examinations were performed in second trimester (median 20.5, range 18.6, 23.3 weeks), and third trimester (median 30.4, range 28.5, 32.9 weeks) (Kooijman et al., 2016). By standardized ultrasound procedures fetal head circumference, abdominal circumference, and femur length were measured (Kooijman et al., 2016; Verburg et al., 2008). The Hadlock formula was used to calculate gestational age adjusted estimated fetal weight and calculate standard deviation scores (SDS) using Dutch growth charts (Hadlock et al., 1985; Verburg et al., 2008).

Sex, expected date of delivery and birth weight was collected from midwives. Sex and gestational age adjusted birth weight SDS were constructed using World Health Organization growth charts (Kiserud et al., 2018). We used gestational age, birth weight and size for gestational age both continuous and categorized into clinically relevant categories. Gestational age was categorized into preterm (<37 weeks), term (37–42 weeks) and postterm (>42 weeks). Birth weight was categorized in low birth weight (<2500 g), normal birth weight (2500–4500 g) and high birth weight (>4500 g). Children with birth weight SDS <10th percentile were classified as small for gestational age (SGA), and those with birth weight SDS >90th percentile were classified as large for gestational age (LGA).

Infant weight was measured at approximately 6 months (median 6.2, range 5.0, 10.0 months), 12 months (median 11.1, range 10.0, 13.0 months), and 24 months of age (median 24.9, range 23.0, 29.0 months). Head circumference was measured between birth and approximately 3 months (median postconceptional age 41.1, range 33.0, 58.4 weeks) and at approximately 12 months (median 11.0, range 10.0, 13.0 months). We created age and sex‐adjusted SDS using Dutch reference growth charts in Growth Analyzer 4.0 (Kooijman et al., 2016).

We constructed individual‐based fetal and infant weight change variables by using estimated fetal weight, age and sex‐adjusted birth weight and infant weight. Fetal weight change was defined as growth in SDS between the second trimester and birth. Infant weight change was defined as growth in SDS from birth to 24 months (*n* = 3097 of *n* = 3891 children). If weight at 24 months was not available, we used weight at 12 months (*n* = 639 of *n* = 3891 children) and if weight at 12 months was not available, we used weight at 6 months (*n* = 155 of *n* = 3891 children). We considered an increase of more than 0.67 SD between time points as growth acceleration and a decrease of more than 0.67 SD between time points as growth deceleration, reflecting the difference between two percentile lines on the growth charts commonly used to define growth deceleration or acceleration in population and clinical research (Cole & Lanham, 2011).

### Emotional, behavioral and cognitive outcomes

At the age of 13 years (range 13.1, 14.7), the primary caregiver (91.3% mothers) was asked to complete the Child Behavior Checklist (CBCL)/6–18. The CBCL is an internationally validated and reliable measure of emotional and behavioral problems (Achenbach & Ruffle, 2000; Hofstra et al., 2002). Based on the behavior of the child in the preceding 6 months, each of the 112 items is rated on a 3‐point scale: 0 (not true), 1 (somewhat true), 2 (very true or often true). Together the 112 items result in the total behavior problems score, a higher score indicating more emotional and behavioral problems. This total behavior problems sum score can be subdivided into the internalizing and externalizing problems scale. Internalizing problems consist of the subscales, emotionally reactive and anxious/depressed symptoms, as well as somatic complaints and symptoms of being withdrawn. Externalizing problems consist of the subscales rule breaking and aggressive behavior (Achenbach & Ruffle, 2000; Blok et al., 2022). The sum scores for the internalizing and externalizing problem scale were derived by summing the raw scores for each respective subscale of which the scale consists. We used the sum score of total behavior problems (*n* = 4133), internalizing problems (*n* = 4136), externalizing problems (*n* = 4125) and of the Diagnostic and Statistical Manual of Mental Disorders (DSM)‐oriented ADHD symptoms (*n* = 4117). We used continuous values and dichotomous cut‐off scores. As in previous analyses conducted in the same study population, a non‐optimal score was defined as the highest 20% and used as the dichotomous cut‐off scores (Cents et al., 2011; Velders et al., 2011). Using the individual‐based CBCL cut‐offs of the 95th and 98th percentile would in our relatively healthy population result in insufficient statistical power Achenbach & Rescorla, 2001).

At the same visit, the primary caregiver also completed an adapted version of the Social Responsiveness Scale, consisting of 18 items. The SRS is a quantitative measure of autistic traits for children 4–18 years old, with higher scores indicating greater social behavior impairment (Lyall et al., 2021; Wagner et al., 2019). The authors of the scale recommend cutoffs for screening consistent with 1.078 for boys and 1.000 for girls (Constantino & Gruber, 2005).

Children’s intelligence quotient (IQ) was assessed using a subset of the Wechsler Intelligence Scale for Children‐Fifth Edition (WISC‐V). The WISC‐V is an instrument assessing cognitive functioning in 6 to 16‐year‐olds. Four core subtests: vocabulary, matrix reasoning, digit span and coding were selected to assess specific cognitive domains and to derive an estimated full scale intelligence quotient (FSIQ) (Kaufman et al., 2016). We used IQ estimates as continuous and dichotomous outcomes, using a cut‐off of <80, as defined in the WISC‐V manual, to distinguish borderline low IQ scores (Kaufman et al., 2016).

For all outcomes, we constructed standard deviation scores ((observed value−mean)/SD) within the population to enable comparison of effect estimates.

A detailed description of the Methods section can be found in the Supplementary Methods.

### Covariates

We used a directed acyclic graphs (DAG) to identify potential covariates (Supplemental Figure S2). We obtained information on maternal age, pre‐pregnancy weight, parity, ethnicity, educational level, smoking, alcohol use and folic acid supplementation using questionnaires and registries during pregnancy (Kooijman et al., 2016). Maternal educational level was self‐reported as the highest completed educational level and categorized as lower (primary plus first 3 years of secondary education), medium (upper secondary and vocational training or management and specialist education) and higher education (associate degree, bachelor or master degree programs). During the 6‐year follow‐up visit we used the 12‐item reliable and validated computerized short version of the Raven’s Progressive Matrices to assess maternal non‐verbal cognitive ability (Raven, 2021).

### Statistical analysis

First, we described maternal, fetal and childhood characteristics. We performed a non‐response analysis by comparing characteristics of children with and without outcome assessments by using Independent Student *T*‐test, Mann‐Whitney U and χ^2^ tests. Second, we used linear and logistic regression models to assess the associations of birth characteristics with continuous and dichotomous scores of behavior and cognitive outcomes at the age of 13 years. We have added scatter plots of the associations to the Supplemental Figures S3‐S5. For all analyses, the continuous scores for total, internalizing and externalizing behavior problems, ADHD symptoms and ASD traits were natural log‐transformed to deal with a skewed distribution. Third, we used estimated fetal weight growth, gestational age adjusted birth weight and infant weight and categorized both fetal weight growth (second trimester to birth) and infant weight growth (birth to 24 months) change each into three groups (growth deceleration, normal growth, and growth acceleration), and created a combined 3 x 3 variable leading to nine different growth patterns. We used multivariable linear or logistic regression models to explore associations of fetal and infant weight changes with continuous and dichotomous behavior and cognitive outcomes. We performed a sensitivity analysis restricting the study population to only children with weight measurements at 24 months (*n* = 3097). Last, to test the robustness of our findings for different growth measures, we performed a sensitivity analysis for fetal and infant head circumference. We categorized head circumference at birth (0–3 months) and during infancy (12 months) into tertiles (smallest, middle and largest) and created a combined variable that reflects 9 different head circumference patterns. Participants were included if they had head circumference data at both time points. Because of the structural difference in the fetal and childhood head circumference reference charts, we did not combine these charts. For all analyses, basic models were adjusted for child’s sex and age at outcome assessment. The confounder‐adjusted model, which we considered the main model, was additionally adjusted for maternal age, parity, pre‐pregnancy body mass index, educational level, ethnicity, prenatal folic acid use, smoking, alcohol use and maternal IQ at 6 years old. Potential confounders were identified based on previous literature and we selected those that fulfilled the graphical criteria for confounding in a DAG and changed the effect estimates >10% after addition to the crude model. Consistently associated with fetal growth, birth weight and infancy weight were maternal educational level, ethnicity, folic acid supplementation, smoking during pregnancy, maternal IQ and BMI at age of outcome measurement. As fetal and infant growth are highly correlated and we considered three groups of outcomes, namely CBCL derivatives, SRS scores and IQ, we took account for multiple testing by specifying significant *p*‐values as *p* < (0.05/3) 0.017. We tested for statistical interaction of maternal educational level, ethnicity, smoking, alcohol use and folic acid supplementation during pregnancy and maternal IQ in these associations but no statistically significant interactions were observed (*p* > 0.05). Missing data in covariates (ranging from 0.4% to 36.9%) were multiple imputed using the Markov Chain Monte Carlo method. Ten imputed datasets were created and analyzed together (Sterne et al., 2009). Statistical analyses were performed using the Statistical Package of Social Sciences version 25.0 for Windows (SPSS Inc., Chicago, IL, USA).

## RESULTS

### Participant characteristics

Table 1 shows the subject characteristics from imputed data. Follow‐up measurements were available for 4716 children with a median age of 13.5 (range 13.1, 14.7) years, of whom 2400 (50.9%) were girls and 2744 (58.2%) were of Dutch ethnicity. Supplementary Table S1 shows subjects characteristics from the observed data. Supplementary Table S2 shows that compared to the study population (*n* = 4716), mothers of children without outcome measurements (*n* = 3906) were younger, less often primipara, of European ethnicity, had lower IQ and lower education, used less alcohol, smoked more often, used folic acid supplements less often, and had higher pre‐pregnancy BMI. Children who were lost to follow‐up were more often male, born preterm and born with a lower birth weight.

**TABLE 1:** Descriptive statistics of the study population.^a^

Characteristic	(*N* = 4716) N (%)
Maternal	
Age at enrollment, median (95% range), years	31.4 (20.2, 39.6)
Pre‐pregnancy BMI, median (95% range), kg/m^2^	22.7 (17.8, 34.1)
Parity nulliparous, N (%)	2757 (58.5)
Education level, N (%)	
Primary education	388 (8.4)
Secondary education	1930 (40.9)
Higher education	2398 (50.7)
Ethnicity, No. (%)	
Dutch	2744 (58.2)
Non‐Dutch, Western	392 (8.3)
Non‐Dutch, Non‐Western	1580 (33.5)
Smoking during pregnancy, N (%)	1108 (23.5)
Alcohol during pregnancy, N (%)	2753 (58.4)
Folic acid supplement use, did not use N (%)	1066 (22.6)
Maternal IQ, mean (SD)	99.8 (18.0)
Fetal	
Second trimester	
Gestational age, median (95% range), weeks	20.5 (18.6, 23.3)
Estimated fetal weight, median (95% range), grams	362 (260, 534)
Third trimester	
Gestational age, median (95% range), weeks	30.4 (28.5, 32.9)
Estimated fetal weight, median (95% range), grams	1602 (1211, 2214)
Birth	
Child sex, female N (%)	2400 (50.9)
Gestational age at birth, median (95% range), weeks	40.1 (35.9, 42.4)
<37 weeks, N (%)	216 (4.6)
37–42 weeks, N (%)	4162 (88.3)
>42 weeks, N (%)	338 (7.2)
Birth weight, median (95% range), grams	3470 (2254, 4500)
<2500 g, N (%)	201 (4.3)
2500–4500 g, N (%)	4388 (93.2)
>4500 g, N (%)	121 (2.6)
Sex and gestational age adjusted birth weight	
Small (<10^th^ percentile), N (%)	471 (10.0)
Appropriate (10^th^ ‐ 90^th^ percentile), N (%)	3768 (80.0)
Large (>90^th^ percentile), N (%)	471 (10.0)
Postconceptional age at head circumference measurement, median (95% range), weeks	41.1 (37.6, 52.9)
Head circumference, mean (SD), cm	35.2 (2.4)
Infant	
At 6‐month visit	
Age at visit, median (95% range), months	6.2 (5.2, 8.3)
Weight, median (95% range), kg	7.8 (6.2, 9.7)
At 12‐month visit	
Age at visit, median (95% range), years	11.1 (10.1, 12.5)
Weight, median (95% range), kg	9.6 (7.6, 11.8)
Head circumference, mean (SD), cm	46.1 (1.4)
At 2‐year visit	
Age at visit, median (95% range), years	24.9 (23.4, 28.2)
Weight, median (95% range), kg	12.8 (10.3, 16.0)
Childhood	
Age at follow‐up, median (95% range), years	13.5 (13.1, 14.7)
CBCL total behavior problems score, median (95% range)	14.0 (0.0, 63.0)
CBCL internalizing problems score, median (95% range)	4.0 (0.0, 21.4)
CBCL externalizing problems score, median (95% range)	2.0 (0.0, 18.0)
CBCL ADHD symptoms score, median (95% range)	2.0 (0.0, 10.0)
SRS autism traits score, median (95% range)	4.0 (0.0, 16.0)
Estimated WISC‐V intelligence quotient, mean (SD)	101.6 (13.9)

*Note*: Values are mean (SD), median (95% range), or number (valid %). WISC‐V.

Abbreviations: ADHD, attention‐deficit hyperactivity disorder; BMI, body mass index; CBCL, Child Behavior Checklist; DSM, Diagnostic and Statistical Manual of Mental Disorders; SRS, Social Responsiveness Scale; WISC‐V, Wechsler Intelligence Scale for Children‐Fifth Edition.

^a^ Characteristics are based on the pooled datasets after multiple imputations.

### Birth characteristics and behavioral and cognitive outcomes

Tables 2 and 3 show the associations of birth characteristics with behavior and cognitive outcomes at 13 years of age (basic models are shown in Supplementary Table S3 and Table S4). One week longer gestational age at birth was associated with a −0.03 SDS (95% Confidence Interval (CI): −0.04, −0.01) lower total behavior problems score, a −0.02 SDS (95% CI: −0.04, −0.01) lower ADHD symptoms score and a lower odds of a low IQ score (Odds Ratio (OR) 0.91 (95% CI: 0.86, 0.97). Compared to children born at term, those born preterm had a 0.20 SDS (95% Confidence Interval (CI) 0.06, 0.35) higher total behavior problems score. An increase in birth weight of 500 g was associated with a −0.04 SDS (95% CI: −0.07, −0.01) lower total behavior problems score, a −0.04 SDS (95% CI: −0.07, −0.01) lower ADHD symptoms score and a 0.06 SDS (95% CI: 0.04, 0.09) higher IQ score. Also an increase in birth weight of 500 g was associated with a lower odds of having high externalizing problems (OR 0.92 (95% CI: 0.86, 0.98), of having high ADHD symptoms (OR 0.91 (95% CI: 0.85, 0.98) and of having a low IQ score (OR 0.79 (95% CI: 0.71, 0.88). A 1‐SDS higher birth weight for gestational age was associated with a 0.07 SDS (95% CI: 0.04, 0.10) higher IQ score and a lower odds of having a low IQ score (OR 0.82 (95% CI: 0.72, 0.94). As compared to children born appropriate size for gestational age (AGA), those born SGA had a 0.13 SDS (95% CI: 0.03, 0.23) higher total behavior problems score, a 0.16 (95% CI: 0.06, 0.27) higher internalizing problems score, a 0.13 SDS (95% CI: 0.03, 0.24) higher ADHD symptoms score and a −0.15 SDS (95% CI: −0.24, −0.05) lower IQ score. In line with these findings, SGA born children had a higher odds of an increased total behavior problems score (OR 1.35 (95% CI: 1.06, 1.72) and internalizing problems score (OR 1.58 [95% CI: 1.26, 1.99]). The associations of preterm birth, postterm birth, low birth weight, and LGA with behavior and cognitive outcomes attenuated into non‐significance after correction for multiple testing.

**TABLE 2:** Associations of birth characteristics with total, internalizing and externalizing behavior problems scores in early adolescence.

		Total problems (*n* = 4133)	Total problems >80^th^ percentile (*n* = 845)^a^	Internalizing problems (*n* = 4136)	Internalizing problems >80^th^ percentile (*n* = 932)^b^	Externalizing problems (*n* = 4125)	Externalizing problems >80^th^ percentile (*n* = 972)^c^
Birth characteristics	N	Difference in SDS (95 CI)	OR (95% CI)	Difference in SDS (95 CI)	OR (95% CI)	Difference in SDS (95 CI)	OR (95% CI)
Gestational age at birth, wk		−0.03 (−0.04 to −0.01)**	0.96 (0.92–1.00)*	−0.02 (−0.04 to −0.00)*	0.99 (0.94–1.03)	−0.02 (−0.04 to −0.00)*	0.95 (0.91–0.99)*
<37	216	0.20 (0.06–0.35)**	1.17 (0.82–1.67)	0.15 (−0.00–0.29)	1.14 (0.81–1.61)	0.10 (−0.05–0.25)	1.18 (0.84–1.66)
37–42	*4162*	*Reference*	*Reference*	*Reference*	*Reference*	*Reference*	*Reference*
>42	338	0.03 (−0.10–0.14)	0.96 (0.71–1.30)	0.01 (−0.11–0.13)	1.00 (0.74–1.34)	0.02 (−0.10–0.14)	0.99 (0.74–1.31)
Birth weight, 500 gr		−0.04 (−0.07 to −0.01)**	0.92 (0.86–0.99)*	−0.03 (−0.06 to −0.01)*	0.93 (0.87–0.99)*	−0.02 (−0.05 to 0.01)	0.92 (0.86–0.98)**
<2500	201	0.15 (0.00–0.30)*	1.15 (0.80–1.65)	0.06 (−0.09–0.22)	1.05 (0.73–1.50)	0.10 (−0.05–0.25)	1.25 (0.88–1.76)
2500–4500	*4388*	*Reference*	*Reference*	*Reference*	*Reference*	*Reference*	*Reference*
>4500	121	0.08 (−0.12–0.27)	0.91 (0.55–1.50)	−0.01 (−0.20 to 0.19)	0.99 (0.61–1.60)	0.14 (−0.05–0.34)	0.88 (0.55–1.41)
Size for gestational age at birth, SD score		−0.02 (−0.05 to 0.02)	0.94 (0.87–1.02)	−0.03 (−0.06 to 0.01)	0.92 (0.86–0.99)*	0.00 (−0.03 to 0.03)	0.94 (0.88–1.02)
Small <10^th^ percentile	471	0.13 (0.03–0.23)**	1.35 (1.06–1.72)**	0.16 (0.06–0.27)**	1.58 (1.26–1.99)**	0.06 (−0.04–0.17)	1.19 (0.94–1.51)
Appropriate 10^th^‐90^th^ percentile	*3768*	*Reference*	*Reference*	*Reference*	*Reference*	*Reference*	*Reference*
Large >90^th^ percentile	470	0.01 (−0.09–0.11)	0.99 (0.77–1.29)	−0.03 (−0.13 to 0.08)	0.87 (0.67–1.12)	0.04 (−0.06–0.15)	0.99 (0.77–1.27)

*Note*: Values are regression coefficients (95% confidence interval) or odds ratios (95% confidence interval) obtained from multivariable linear respectively logistic regression models and reflect the differences in parent‐reported behavioral problems, the total problems (SDS), internalizing problems (SDS) and externalizing problems (SDS) for birth characteristics. For the logistic regressions the top 20% of the total score, the internalizing and the externalizing problem score was used. Pooled estimates are from multiple imputed datasets. The confounder model is adjusted for age at time of measurement and sex of the child, maternal age in pregnancy, parity, pre‐pregnancy body mass index, educational level, ethnicity, folic acid use, smoking, alcohol and maternal IQ measured at the child age of 6 years old.

Abbreviations: CI, confidence interval, OR, odds ratio; SDS, standard deviation score; wk, weeks.

Total number of available cases; ^a^: *n* = 4133, ^b^: *n* = 4136, ^c^: *n* = 4125.

*p value < 0.05, **p value < 0.017.

**TABLE 3:** Associations of birth characteristics with ADHD symptoms and ASD traits score and IQ in early adolescence.

		ADHD symptoms score (*n* = 4117)	ADHD symptoms score >80^th^ percentile (*n* = 1009)^a^	Autistic traits score (*n* = 4130)	Autistic traitsscore cut‐off (*n* = 362)^b^	IQ score (*n* = 4292)	IQ score <80 (*n* = 261)^c^
Birth characteristics	N	Difference in SDS (95 CI)	OR (95% CI)	Difference in SDS (95 CI)	OR (95% CI)	Difference in SDS (95 CI)	OR (95% CI)
Gestational age at birth, wk		−0.02 (−0.04 to −0.01)**	0.96 (0.92–0.99)*	−0.02 (−0.03 to 0.00)	0.95 (0.90–1.01)	0.02 (0.00–0.03)*	0.91 (0.86–0.97)**
<37	216	0.18 (0.03–0.32)*	1.31 (0.94–1.82)	0.03 (−0.12–0.18)	1.16 (0.71–1.90)	−0.04 (−0.18 to 0.09)	1.70 (1.03–2.81)*
37–42	*4162*	*Reference*	*Reference*	*Reference*	*Reference*	*Reference*	*Reference*
>42	338	−0.01 (−0.13 to 0.10)	0.96 (0.72–1.27)	−0.12 (−0.24 to −0.00)*	0.94 (0.61–1.45)	0.11 (−0.00–0.21)	0.87 (0.50–1.51)
Birth weight, 500 gr		−0.04 (−0.07 to −0.01)**	0.91 (0.85–0.98)**	−0.03 (−0.05 to 0.00)	0.91 (0.83–1.01)	0.06 (0.04–0.09)**	0.79 (0.71–0.88)**
<2500	201	0.18 (0.03–0.33)*	1.33 (0.94–1.87)	0.08 (−0.07–0.23)	1.49 (0.93–2.39)	−0.06 (−0.20 to 0.08)	1.79 (1.07–2.99)*
2500–4500	*4388*	*Reference*	*Reference*	*Reference*	*Reference*	*Reference*	*Reference*
>4500	121	0.04 (−0.16–0.23)	0.84 (0.53–1.35)	−0.01 (−0.20 to 0.18)	0.70 (0.32–1.53)	0.13 (−0.05–0.31)	0.52 (0.18–1.47)
Size for gestational age at birth, SD score		−0.02 (−0.05 to 0.01)	0.94 (0.87–1.01)	−0.02 (−0.05 to 0.01)	0.94 (0.84–1.06)	0.07 (0.04–0.10)**	0.82 (0.72–0.94)**
Small <10^th^ percentile	471	0.13 (0.03–0.24)**	1.22 (0.96–1.54)	0.07 (−0.04–0.17)	1.18 (0.84–1.66)	−0.15 (−0.24 to −0.05)**	1.47 (1.00–2.15)*
Appropriate 10^th^‐90^th^ percentile	*3768*	*Reference*	*Reference*	*Reference*	*Reference*	*Reference*	*Reference*
Large >90^th^ percentile	470	−0.01 (−0.11 to 0.09)	0.95 (0.74–1.21)	−0.01 (−0.11 to 0.10)	0.85 (0.57–1.26)	0.11 (0.02–0.21)*	1.07 (0.67–1.71)

*Note*: Values are regression coefficients (95% confidence interval) or odds ratio’s (95% confidence interval) obtained from multivariable linear respectively logistic regression models and reflect the differences in parent‐reported behavioral problems, ADHD symptoms score (SDS), autistic traits score (SDS) and IQ (SDS) for birth characteristics. For the logistic regressions the top 20% of the ADHD symptoms score, autistic traits score (weighted scores of >1.078 for boys and >1.000 for girls) and IQ (score <80) was used. Pooled estimates are from multiple imputed datasets. The confounder model is adjusted for age at time of measurement and sex of the child, maternal age in pregnancy, parity, pre‐pregnancy body mass index, educational level, ethnicity, folic acid use, smoking, alcohol and maternal IQ measured at the child age of 6 years old.

Abbreviations: ADHD, attention‐deficit hyperactivity disorder; CI, confidence interval; IQ, intelligence quotient; OR, odds ratio; SDS, standard deviation score.

Total number of available cases; ^a^: *n* = 4117, ^b^: *n* = 4130, ^c^: *n* = 4292.

*p value < 0.05, **p value < 0.017.

### Fetal and infant growth and behavioral and cognitive outcomes

Tables 4 and 5 show that as compared to children with normal fetal and infant growth, those with fetal growth deceleration, followed by normal infant growth had at age 13 years decreased odds of a high total behavior problems score (OR 0.66 [95% CI: 0.48, 0.91]). Children with fetal growth acceleration followed by normal infant growth had a −0.15 SDS (95% CI: −0.26, −0.03) lower total behavior problems score and a lower odds of having a high total behavior problems score (OR 0.65 (95% CI: 0.48, 0.88). Children with fetal and infant growth acceleration had the highest IQ score (difference with reference group 0.27 SDS [95% CI: 0.11, 0.44]). The following associations of fetal and infant growth patterns with ASD traits and IQ attenuated into non‐significance after correction for multiple testing was applied. Children with fetal growth acceleration followed by infant growth deceleration also had lower odds of ASD traits above the screening cut‐off (OR 0.62 [95% CI: 0.39, 0.99]). Children with normal fetal growth followed by infant growth acceleration had lower odds of ASD traits above the screening cut‐off (OR 0.58 [95% CI: 0.34–0.96]). Children with fetal growth deceleration followed by infant growth acceleration had lower odds of a low IQ score (OR: 0.48 [95% CI: 0.25, 0.93]). The corresponding basic models are shown in Supplementary Tables S5 and S6. The results were largely similar when we restricted the analyses for the associations of fetal and infant growth patterns with behavioral and cognitive outcomes to only children with weight measurements at 24 months (Supplementary Table S9 and S10). Supplementary Table S11 shows the distribution of different fetal and infant growth patterns in children born preterm, with low birth weight and small for gestational age.

**TABLE 4:** Associations of fetal and infant growth patterns with total, internalizing and externalizing behavior problems scores in early adolescence.

		Total problems (*n* = 3315)	Total problems >80^th^ percentile (*n* = 644)^a^	Internalizing problems (*n* = 3318)	Internalizing problems >80^th^ percentile (*n* = 723)^b^	Externalizing problems (*n* = 3309)	Externalizing problems >80^th^ percentile (*n* = 747)^c^
Fetal and infant growth patterns	N	Difference in SDS (95 CI)	OR (95% CI)	Difference in SDS (95 CI)	OR (95% CI)	Difference in SDS (95 CI)	OR (95% CI)
Fetal growth deceleration							
Infant growth deceleration	129	0.04 (−0.15–0.24)	0.90 (0.56–1.47)	0.07 (−0.12–0.27)	0.83 (0.51–1.35)	−0.12 (−0.31 to 0.08)	0.72 (0.44–1.18)
Infant normal growth	408	−0.06 (−0.18 to 0.06)	0.66 (0.48–0.91)**	−0.01 (−0.14 to 0.11)	0.78 (0.57–1.06)	−0.10 (−0.22 to 0.03)	0.85 (0.63–1.14)
Infant growth acceleration	419	−0.03 (−0.15 to 0.10)	0.82 (0.60–1.12)	0.01 (−0.12–0.13)	0.92 (0.68–1.24)	−0.01 (−0.13 to 0.12)	0.92 (0.68–1.24)
Fetal normal growth							
Infant growth deceleration	351	0.02 (−0.11–0.15)	0.93 (0.68–1.28)	0.02 (−0.11–0.15)	1.02 (0.75–1.39)	0.02 (−0.11–0.15)	1.01 (0.74–1.37)
Infant normal growth	*869*	*Reference*	*Reference*	*Reference*	*Reference*	*Reference*	*Reference*
Infant growth acceleration	398	−0.06 (−0.19 to 0.07)	0.69 (0.50–0.97)*	−0.01 (−0.13 to 0.12)	0.85 (0.63–1.16)	−0.06 (−0.19 to 0.06)	0.74 (0.54–1.02)
Fetal growth acceleration							
Infant growth deceleration	479	0.02 (−0.10–0.13)	0.77 (0.57–1.05)	−0.03 (−0.15 to 0.09)	0.85 (0.64–1.14)	0.01 (−0.11–0.12)	0.80 (0.60–1.07)
Infant normal growth	505	−0.15 (−0.26 to −0.03)**	0.65 (0.48–0.88)**	−0.13 (−0.24 to −0.01)*	0.78 (0.59–1.04)	−0.10 (−0.21 to 0.02)	0.77 (0.58–1.03)
Infant growth acceleration	150	−0.09 (−0.28 to 0.09)	0.82 (0.51–1.30)	−0.07 (−0.25 to 0.11)	0.74 (0.47–1.18)	−0.03 (−0.21 to 0.16)	0.77 (0.49–1.22)

*Note*: Values are regression coefficients (95% confidence interval) or odds ratio’s (95% confidence interval) obtained from multivariable linear respectively logistic regression models and reflect the differences in parent‐reported behavioral problems, the total problems (SDS), internalizing problems (SDS) and externalizing problems (SDS) for fetal and infant growth patterns. For the logistic regressions the top 20% of the total problems, the internalizing and the externalizing problems was used. Pooled estimates are from multiple imputed datasets. The confounder model is adjusted for age at time of measurement and sex of the child, maternal age in pregnancy, parity, pre‐pregnancy body mass index, educational level, ethnicity, folic acid use, smoking, alcohol and maternal IQ measured at the child age of 6 years old.

Abbreviations: CI, confidence interval; OR, odds ratio; SDS, standard deviation score.

Total number of available cases; ^a^: *n* = 3315, ^b^: *n* = 3318, ^c^: *n* = 3309.

*p value < 0.05, **p value < 0.017.

**TABLE 5:** Associations of fetal and infant growth patterns with ADHD symptoms, autism traits and IQ in early adolescence.

		ADHD symptoms score (*n* = 3304)	ADHD symptoms score >80^th^ percentile (*n* = 764)^a^	Autistic traits score (*n* = 3315)	Autistic traits score cut‐off (*n* = 274)^b^	IQ score (*n* = 3348)	IQ score cut off <80 (*n* = 171)^c^
Fetal and infant growth patterns	N	Difference in SDS (95 CI)	OR (95% CI)	Difference in SDS (95 CI)	OR (95% CI)	Difference in SDS (95 CI)	OR (95% CI)
Fetal growth deceleration							
Infant growth deceleration	129	−0.05 (−0.24 to 0.14)	0.93 (0.58–1.49)	−0.10 (−0.29 to 0.10)	0.62 (0.28–1.34)	−0.05 (−0.23 to 0.13)	1.95 (0.95–4.01)
Infant normal growth	408	0.04 (−0.08–0.16)	0.99 (0.74–1.32)	−0.00 (−0.12 to 0.12)	0.84 (0.54–1.31)	−0.07 (−0.18 to 0.05)	1.17 (0.69–1.97)
Infant growth acceleration	419	−0.02 (−0.14 to 0.11)	0.99 (0.73–1.33)	−0.09 (−0.21 to 0.04)	0.82 (0.52–1.29)	0.04 (−0.08–0.15)	0.48 (0.25–0.93)*
Fetal normal growth							
Infant growth deceleration	351	−0.00 (−0.13 to 0.12)	0.80 (0.58–1.10)	−0.01 (−0.14 to 0.12)	1.05 (0.68–1.63)	0.08 (−0.04–0.20)	0.70 (0.37–1.35)
Infant normal growth	*869*	*Reference*	*Reference*	*Reference*	*Reference*	*Reference*	*Reference*
Infant growth acceleration	398	−0.05 (−0.17 to 0.08)	0.83 (0.61–1.14)	−0.05 (−0.18 to 0.08)	0.58 (0.34–0.96)*	0.10 (−0.02–0.21)	0.87 (0.50–1.53)
Fetal growth acceleration							
Infant growth deceleration	479	0.07 (−0.05–0.19)	0.94 (0.71–1.25)	−0.07 (−0.19 to 0.05)	0.62 (0.39–0.99)*	0.09 (−0.02–0.20)	0.92 (0.52–1.62)
Infant normal growth	505	−0.09 (−0.20 to 0.02)	0.85 (0.64–1.13)	−0.08 (−0.19 to 0.04)	0.95 (0.63–1.43)	0.08 (−0.03–0.19)	0.84 (0.48–1.47)
Infant growth acceleration	150	−0.14 (−0.32 to 0.04)	0.83 (0.53–1.32)	−0.07 (−0.25 to 0.12)	0.83 (0.43–1.62)	0.27 (0.11–0.44)**	0.52 (0.20–1.36)

*Note*: Values are regression coefficients (95% confidence interval) or odds ratio’s (95% confidence interval) obtained from multivariable linear respectively logistic regression models and reflect the differences in parent‐reported behavioral problems, ADHD symptoms score (SDS), autistic traits score (SDS) and IQ (SDS) for fetal and infant growth patterns. For the logistic regressions the top 20% of the ADHD symptoms score, autistic traits score (weighted scores of >1.078 for boys and >1.000 for girls) and IQ (score <80) was used. Pooled estimates are from multiple imputed datasets. The confounder model is adjusted for age at time of measurement and sex of the child, maternal age in pregnancy, parity, pre‐pregnancy body mass index, educational level, ethnicity, folic acid use, smoking, alcohol and maternal IQ measured at the child age of 6 years old.

Abbreviations: ADHD, attention‐deficit hyperactivity disorder; CI, confidence interval; IQ, intelligence quotient; OR, odds ratio; SDS, standard deviation score.

Total number of available cases; ^a^: *n* = 3304, ^b^: *n* = 3315, ^c^: *n* = 3348.

*p value < 0.05, **p value < 0.017.

### Birth and infant head circumference and childhood outcomes

Supplementary Tables S7 and S8 show the associations of birth and infant head circumference patterns with emotional, behavioral and cognitive outcomes at age 13 years. Children with a head circumference in the smallest tertile at birth and in infancy had a 0.26 SDS (95% CI: 0.10, 0.42) higher total behavior problems score, a 0.24 SDS (95% CI: 0.07, 0.40) higher externalizing problems score and higher odds of a high ADHD symptoms score (OR: 1.77 [95% CI: 1.22, 2.57]) and low IQ score (OR: 2.64 [95% CI: 1.48–4.70]). Overall the associations were similar in direction but stronger for birth and infant head circumference patterns than for fetal and infant weight growth patterns.

## DISCUSSION

In this population‐based prospective cohort study, we observed that a longer gestational age at birth was associated with lower parent‐reported scores for total behavior problems and ADHD symptoms in early adolescence. Increased birth weight was associated with lower odds of parent‐reported high externalizing problems and ADHD symptoms, and lower odds of a low IQ. Children born SGA had higher parent‐reported total and internalizing behavior problems, ADHD symptoms, and lower IQ in early adolescence, compared to those born AGA. Children with accelerated fetal and infant growth had a higher IQ in early adolescence, whereas those with decelerated fetal growth followed by normal infant growth had lower odds of a high parent‐reported total behavior problems score. The associations of fetal and infant head circumference with behavior and cognitive outcomes were stronger than those of fetal and infant weight growth patterns with the same behavioral and cognitive outcomes.

Small size for gestational age, low birth weight and prematurity are associated with increased risks of impaired cognitive development, behavioral problems, psychopathology and intellectual disabilities (Anderson et al., 2021; El Marroun et al., 2012; Grootendorst‐van Mil et al., 2015; Hack et al., 2005; Johnson & Marlow, 2011). In a large meta‐analysis, 81% of studies showed increased internalizing and externalizing problems in preterm born children (Bhutta et al., 2002). The estimated prevalence’s of behavioral problems depends on the severity of prematurity and low birth weight and range from 13% to 46% (Johnson & Marlow, 2011). We observed that preterm birth and being born SGA were associated with higher scores for total behavior problems, being born SGA was also associated with higher scores for internalizing problems in children aged 13 years. Also, a longer gestational duration and higher birth weight were associated with lower scores for total behavior problems. Longitudinal growth analyses suggest that children who experienced fetal growth deceleration or acceleration followed by normal infant growth had lower odds of high total behavior problems. Thus, our results suggest that next to birth characteristics, patterns of fetal and infant body growth might be important for the risks of behavioral problems in early adolescence.

Previous studies suggest that children born preterm or SGA are at higher risk of ADHD (Allotey et al., 2018; Anderson et al., 2021; Bhutta et al., 2002; Cortese et al., 2021; Pettersson et al., 2019; van Mil et al., 2015). A previous study conducted in Canada among 274 children showed that children who were fetal growth restricted but showed childhood catch‐up growth had increased impulsivity at 5 years of age, as compared to normal birth weight children (Silveira et al., 2018). Also, results from a previous study in the same study population as the current, showed that those who were small from mid pregnancy until birth had slightly more ADHD symptoms at 6 year old, as compared to children appropriate size for gestational age (Ferguson et al., 2021). In line with previous studies, our results suggest that older gestational age and higher weight at birth are associated with lower ADHD symptoms, whereas being born SGA is associated with higher ADHD symptoms at 13 years old. However, we did not observe any association between fetal and infant weight growth patterns and ADHD in childhood. The differences in results might be explained by the differences in underlying populations and different approaches to assess growth.

A previous international cohort study of more than 3,500,000 individuals, including over 50,000 individuals with ASD, showed that both preterm and postterm birth are risk factors for ASD (Persson et al., 2020). In addition to gestational age at birth, also early life growth might be related to ASD (Dhaliwal et al., 2019). One Japanese case‐control study with 280 ASD cases and 609 controls reported that children with ASD were heavier in infancy (Yang et al., 2011). We observed lower ASD traits in postterm born children as compared to term born. Also, we observed a lower odds of ASD traits above the screenings cut‐off in children with fetal normal growth followed by infant growth acceleration and children with fetal growth acceleration followed by infant deceleration. However, these results attenuated into non‐significance after correction for multiple testing was applied. Previous studies seem to suggest that gestational age on both ends of the spectrum is associated with ASD in childhood (Persson et al., 2020). Children with ASD might follow a different growth pattern in fetal life and infancy (Dhaliwal et al., 2019; Yang et al., 2011). However, from our study we cannot conclude that gestational age or a specific early life growth pattern is associated with ASD traits.

Childhood and adulthood IQ tend to be lower in individuals born preterm or with low birth weight (Cortese et al., 2021; Ferguson et al., 2021; Grootendorst‐van Mil et al., 2015; Kirkegaard et al., 2020; Sacchi et al., 2020). A study conducted in Belarus among 11,899 healthy singletons observed positive associations of increased birth weight and increased childhood weight gain with IQ (Yang et al., 2011). We observed lower IQ for children born SGA. In contrast, longer gestational age, higher birth weight, and size for gestational age were all positively associated with IQ. Additionally, we found the highest IQ in early adolescence among children with fetal and infant growth acceleration. These findings suggest that higher fetal and infant growth are important for cognitive development in later life.

Altogether, we observed that preterm birth and lower weight gain in both fetal life and infancy seem to be related to unfavorable behavioral and cognitive outcomes. To ensure sufficient power in our analyses our main results are based on fetal and infant weight growth. However, sensitivity analyses on early life head circumference growth showed associations of similar direction but stronger than the observed associations of early life weight growth with behavioral and cognitive outcomes. This suggests that head circumference in itself is a growth parameter of great value which could be a proxy for brain development in these associations. In previous research, we have reported that increased weight gain during the fetal period and in infancy is associated with larger brain volumes on MRI scans at 10 years of age, suggesting that brain development is reflected by head circumference and weight growth in fetal life and infancy (Silva et al., 2021). Furthermore, previous research has shown that larger head circumference at birth and in infancy is associated with higher adult IQ and that this association is even stronger in children born very preterm or very low birth weight (Jaekel et al., 2019). Both fetal life and infancy are characterized by rapid body and brain growth. During the fetal life rapid maturation of the fetal brain occurs. Between a gestational age of 12–20 weeks, neuronal migration takes place and around 34 weeks of gestation approximately 40,000 synapses are being formed every second (Monk et al., 2019). This process continues in early childhood. Fetal and infant growth might be explained by various environmental and genetic factors, which might both also affect brain development. Previous studies have shown that growth acceleration following low birth weight of SGA can be both beneficial and detrimental (Ong, 2007). Interestingly, the beneficial effects of growth acceleration seem to be cognitive development whereas the detrimental effects seem to be related to the risk of obesity and adverse metabolic health (Ong, 2007). Previous research has shown the importance of fetal development and reaching growth potential during pregnancy, but has also shown that this potential is dependent on genetic and environmental factors making it difficult to define optimal individual growth potential (Grantz et al., 2018). Especially since excessive childhood weight gain is associated with many childhood and later life complications, such as higher risk of cardiovascular and metabolic diseases (Aggoun, 2007; Goncalves et al., 2022). Although, the observed effects are small, our results also suggest that optimizing growth and development from early fetal life onwards might be important for childhood brain, behavior and cognitive development on a population‐based level. To balance optimal growth versus inadequate or excessive weight gain, individualized growth patterns related to different health outcomes would be most suited. Unfortunately, these have not yet been developed and further research should be focused on identification of such growth patterns.

Strengths of this study include the study design, large number of participants, detailed data on weight measurements from second trimester up to 2 years of age and extensive report on behavioral and cognitive outcomes. In addition to previous research conducted in our study population examining similar associations, this study is of added value since it is conducted in older children and adds fetal‐infant growth patterns. This study also has limitations. Of the 8624 singleton live births with information on fetal or infant growth, 4716 children had data regarding behavior and cognitive outcome measurements. Mothers of children not included in our analyses were younger, less often primipara, higher educated and of European ethnicity, had lower IQ and less often used alcohol during pregnancy. This seems to suggest bias to a relatively more healthy population and might affect the generalizability of our results. Previous research has shown that the association of birth weight and gestational age at birth with IQ or behavior in later life might not be linear. The positive effect of gestational age on IQ seems greater below 34 weeks of gestation as compared to 34–37 weeks of gestation (Eves et al., 2023; MacKay et al., 2010; Wolke et al., 2015). In our population the number of preterm born children is small (4.6%) and an even smaller number of those born preterm was below <34 weeks (1.1%). Therefore we did not have enough numbers to stratify on gestational age or even on term birth versus preterm birth. Such stratification might be relevant for studies with larger numbers since rapid infant weight gain may be stronger associated with neurodevelopmental benefits for preterm infants than term infants (Belfort et al., 2010). Furthermore, the measurement of estimated fetal weight can be inaccurate and random error might be high, especially in the extreme values of estimated fetal weight (Dudley, 2005; Ewington et al., 2024). The stronger effects of postnatal weight growth than fetal weight growth might be due to biological effects or the results of better precision for postnatal measurements. We were interested in the combination between fetal and infant growth delta’s, which are in line with clinical guidelines regarding growth curves and the cut‐offs used for growth deceleration and acceleration respectively. Further studies might focused on more detailed growth modeling aiming to identify the optimal growth patterns in early life for mental health outcomes. Although we adjusted for a large number of potential confounders, residual confounding might still be a possibility due to the observational nature of the study.

## CONCLUSION

In a population‐based sample of children followed from fetal life to adolescence, both gestational age at birth and fetal and infant growth were associated with behavioral and cognitive outcomes in early adolescence. Identification of healthy growth patterns is an important step, which might provide an opportunity in preventing psychopathology. Follow‐up studies are needed to assess the underlying mechanisms and long term consequences of the observed associations.

## AUTHOR CONTRIBUTIONS

**Romy Gonçalves**: Conceptualization; Data curation; Formal analysis; Investigation; Methodology; Project administration; Validation; Visualization; Writing – original draft; Writing – review & editing. **Romy Gaillard**: Conceptualization; Funding acquisition; Supervision; Validation; Writing – review & editing. **Kelly Ferguson**: Funding acquisition; Methodology; Validation; Writing – review & editing. **Sara Sammallahti**: Funding acquisition; Validation; Writing – review & editing. **Manon Hillegers**: Funding acquisition; Methodology; Validation; Writing – review & editing. **Eric Steegers**: Funding acquisition; Methodology; Supervision; Validation; Visualization; Writing – review & editing. **Hanan El Marroun**: Conceptualization; Methodology; Visualization; Writing – review & editing. **Vincent Jaddoe**: Conceptualization; Funding acquisition; Methodology; Supervision; Writing – review & editing.

## CONFLICT OF INTEREST STATEMENT

The authors have declared no competing or potential conflicts of interest.

## ETHICAL CONSIDERATIONS

Nil.

## Supporting information

Supporting Information S1

## Data Availability

Data from the Generation R study are available upon request to the director (generationr@erasmusmc.nl), subject to local rules and regulations.

## References

[jcv212278-bib-0001] Achenbach, T. M. , & Rescorla, L. A. (2001). Manual for the ASEBA school‐age forms & profiles: An integrated system of multi‐informant assessment.

[jcv212278-bib-0002] Achenbach, T. M. , & Ruffle, T. M. (2000). The Child Behavior Checklist and related forms for assessing behavioral/emotional problems and competencies. Pediatr Rev, 21(8), 265–271. 10.1542/pir.21-8-265 10922023

[jcv212278-bib-0003] Aggoun, Y. (2007). Obesity, metabolic syndrome, and cardiovascular disease. Pediatr Res, 61(6), 653–659. 10.1203/pdr.0b013e31805d8a8c 17426660

[jcv212278-bib-0004] Allotey, J. , Zamora, J. , Cheong‐See, F. , Kalidindi, M. , Arroyo‐Manzano, D. , Asztalos, E. , Van der Post, J. , Mol, B. W. , Moore, D. , Birtles, D. , Khan, K. S. , & Thangaratinam, S. (2018). Cognitive, motor, behavioural and academic performances of children born preterm: A meta‐analysis and systematic review involving 64 061 children. BJOG, 125(1), 16–25. 10.1111/1471-0528.14832 29024294

[jcv212278-bib-0005] Anderson, P. J. , De Miranda, D. M. , Albuquerque, M. R. , Indredavik, M. S. , Evensen, K. A. I. , Van Lieshout, R. , Saigal, S. , Taylor, H. G. , Raikkonen, K. , Kajantie, E. , Marlow, N. , Johnson, S. , Woodward, L. J. , Austin, N. , Nosarti, C. , Jaekel, J. , Wolke, D. , Cheong, J. L. , Burnett, A. , & Doyle, L. W. (2021). Psychiatric disorders in individuals born very preterm / very low‐birth weight: An individual participant data (IPD) meta‐analysis. EClinicalMedicine, 42, 101216. 10.1016/j.eclinm.2021.101216 34901794 PMC8639417

[jcv212278-bib-0006] Bach, C. C. , Henriksen, T. B. , Larsen, R. T. , Aagaard, K. , & Matthiesen, N. B. (2020). Head circumference at birth and school performance: A nationwide cohort study of 536,921 children. Pediatr Res, 87(6), 1112–1118. 10.1038/s41390-019-0683-2 31779026

[jcv212278-bib-0007] Belfort, M. B. , Martin, C. R. , Smith, V. C. , Gillman, M. W. , & McCormick, M. C. (2010). Infant weight gain and school‐age blood pressure and cognition in former preterm infants. Pediatrics, 125(6), e1419–e1426. 10.1542/peds.2009-2746 20478940 PMC4138041

[jcv212278-bib-0008] Bhutta, A. T. , Cleves, M. A. , Casey, P. H. , Cradock, M. M. , & Anand, K. J. (2002). Cognitive and behavioral outcomes of school‐aged children who were born preterm: A meta‐analysis. JAMA, 288(6), 728–737. 10.1001/jama.288.6.728 12169077

[jcv212278-bib-0009] Blok, E. , Schuurmans, I. K. , Tijburg, A. J. , Hillegers, M. , Koopman‐Verhoeff, M. E. , Muetzel, R. L. , Tiemeier, H. , & White, T. (2022). Cognitive performance in children and adolescents with psychopathology traits: A cross‐sectional multicohort study in the general population. Dev Psychopathol, 35(2), 1–15. 10.1017/S0954579422000165 35249585

[jcv212278-bib-0010] Cents, R. A. , Tiemeier, H. , Luijk, M. P. , Jaddoe, V. W. , Hofman, A. , Verhulst, F. C. , & Lambregtse‐van den Berg, M. P. (2011). Grandparental anxiety and depression predict young children’s internalizing and externalizing problems: the generation R study. J Affect Disord, 128(1–2), 95–105. 10.1016/j.jad.2010.06.020 20637508

[jcv212278-bib-0011] Cole, S. Z. , & Lanham, J. S. (2011). Failure to thrive: An update. Am Fam Physician, 83(7), 829–834. d8783. 21524049

[jcv212278-bib-0012] Constantino, J. N. , & Gruber, C. P. (2005). Social Responsiveness scale (SRS).

[jcv212278-bib-0013] Cortese, M. , Moster, D. , & Wilcox, A. J. (2021). Term birth weight and neurodevelopmental outcomes. Epidemiology, 32(4), 583–590. 10.1097/EDE.000000000000135000001648-202107000-00016 34001752 PMC8439103

[jcv212278-bib-0014] Dhaliwal, K. K. , Orsso, C. E. , Richard, C. , Haqq, A. M. , & Zwaigenbaum, L. (2019). Risk factors for unhealthy weight gain and obesity among children with autism spectrum disorder. Int J Mol Sci, 20(13), 3285. 10.3390/ijms20133285 31277383 PMC6650879

[jcv212278-bib-0015] Dudley, N. J. (2005). A systematic review of the ultrasound estimation of fetal weight. Ultrasound Obstet Gynecol, 25(1), 80–89. 10.1002/uog.1751 15505877

[jcv212278-bib-0016] El Marroun, H. , Zeegers, M. , Steegers, E. A. , Van der Ende, J. , Schenk, J. J. , Hofman, A. , Jaddoe, V. W. , Verhulst, F. C. , & Tiemeier, H. (2012). Post‐term birth and the risk of behavioural and emotional problems in early childhood. Int J Epidemiol, 41(3), 773–781. 10.1093/ije/dys043 22552873

[jcv212278-bib-0017] Eves, R. , Wolke, D. , Spiegler, J. , & Lemola, S. (2023). Association of birth weight centiles and gestational age with cognitive performance at age 5 years. JAMA Netw Open, 6(8), e2331815. 10.1001/jamanetworkopen.2023.31815 37651137 PMC10472194

[jcv212278-bib-0018] Ewington, L. J. , Hugh, O. , Butler, E. , Quenby, S. , & Gardosi, J. (2024). Accuracy of antenatal ultrasound in predicting large‐for‐gestational‐age babies: Population‐based cohort study. Am J Obstet Gynecol. 10.1016/j.ajog.2024.04.052 38723984

[jcv212278-bib-0019] Ferguson, K. K. , Sammallahti, S. , Rosen, E. , Van den Dries, M. , Pronk, A. , Spaan, S. , Guxens, M. , Tiemeier, H. , Gaillard, R. , & Jaddoe, V. W. V. (2021). Fetal growth trajectories among small for gestational age babies and child neurodevelopment. Epidemiology, 32(5), 664–671. 10.1097/EDE.000000000000138700001648-202109000-00008 34086648 PMC8338787

[jcv212278-bib-0020] Geraedts, E. J. , Van Dommelen, P. , Caliebe, J. , Visser, R. , Ranke, M. B. , Van Buuren, S. , Wit, J. M. , & Oostdijk, W. (2011). Association between head circumference and body size. Horm Res Paediatr, 75(3), 213–219. 10.1159/000321192 21311161

[jcv212278-bib-0021] Goncalves, R. , Wiertsema, C. J. , Silva, C. C. V. , Monasso, G. S. , Gaillard, R. , Steegers, E. A. P. , Santos, S. , & Jaddoe, V. W. V. (2022). Associations of fetal and infant growth patterns with early markers of arterial health in school‐aged children. JAMA Netw Open, 5(6), e2219225. 10.1001/jamanetworkopen.2022.19225 35767260 PMC9244605

[jcv212278-bib-0022] Grantz, K. L. , Hediger, M. L. , Liu, D. , & Buck Louis, G. M. (2018). Fetal growth standards: The NICHD fetal growth study approach in context with INTERGROWTH‐21st and the World health organization multicentre growth reference study. Am J Obstet Gynecol, 218(2S), S641–S655 e628. 10.1016/j.ajog.2017.11.593 29275821 PMC5807181

[jcv212278-bib-0023] Grootendorst‐van Mil, N. H. , Verhulst, F. C. , & Tiemeier, H. (2015). [The association of gestational duration and birth weight with child IQ: The Generation R Study] Prenatale invloeden en neuro‐ontwikke‐ling: associatie van zwangerschapsduur en geboortegewicht met non‐verbaal IQ in de Generation R Studie. Tijdschr Psychiatr, 57(12), 886–891. TVPart_10725. 26727564

[jcv212278-bib-0024] Hack, M. , Taylor, H. G. , Drotar, D. , Schluchter, M. , Cartar, L. , Andreias, L. , Wilson‐Costello, D. , & Klein, N. (2005). Chronic conditions, functional limitations, and special health care needs of school‐aged children born with extremely low‐birth‐weight in the 1990s. JAMA, 294(3), 318–325. 10.1001/jama.294.3.318 16030276

[jcv212278-bib-0025] Hadlock, F. P. , Harrist, R. B. , Sharman, R. S. , Deter, R. L. , & Park, S. K. (1985). Estimation of fetal weight with the use of head, body, and femur measurements‐‐a prospective study. Am J Obstet Gynecol, 151(3), 333–337. 10.1016/0002-9378(85)90298-4 3881966

[jcv212278-bib-0026] Hofstra, M. B. , Van der Ende, J. , & Verhulst, F. C. (2002). Child and adolescent problems predict DSM‐IV disorders in adulthood: A 14‐year follow‐up of a Dutch epidemiological sample. J Am Acad Child Adolesc Psychiatry, 41(2), 182–189. 10.1097/00004583-200202000-00012 11837408

[jcv212278-bib-0027] Jaekel, J. , Sorg, C. , Baeuml, J. , Bartmann, P. , & Wolke, D. (2019). Head growth and intelligence from birth to adulthood in very preterm and term born individuals. J Int Neuropsychol Soc, 25(1), 48–56. 10.1017/S135561771800084X 30426909

[jcv212278-bib-0028] Johnson, S. , & Marlow, N. (2011). Preterm birth and childhood psychiatric disorders. Pediatr Res, 69(5 Pt 2), 11R–18R. 10.1203/PDR.0b013e318212faa0 21289534

[jcv212278-bib-0029] Kaufman, A. S. E. R. , Susan, C. , & Diane, L. (2016). Intelligent testing with the WISC‐V. John Wiley & Sons.

[jcv212278-bib-0030] Kirkegaard, H. , Moller, S. , Wu, C. , Haggstrom, J. , Olsen, S. F. , Olsen, J. , & Nohr, E. A. (2020). Associations of birth size, infancy, and childhood growth with intelligence quotient at 5 years of age: A Danish cohort study. Am J Clin Nutr, 112(1), 96–105. 10.1093/ajcn/nqaa051 32232408 PMC7326594

[jcv212278-bib-0031] Kiserud, T. , Benachi, A. , Hecher, K. , Perez, R. G. , Carvalho, J. , Piaggio, G. , & Platt, L. D. (2018). The World health organization fetal growth charts: Concept, findings, interpretation, and application. Am J Obstet Gynecol, 218(2S), S619–S629. 10.1016/j.ajog.2017.12.010 29422204

[jcv212278-bib-0032] Konkel, L. (2018). The brain before birth: Using fMRI to explore the secrets of fetal neurodevelopment. Environ Health Perspect, 126(11), 112001. 10.1289/EHP2268 30457876 PMC6371691

[jcv212278-bib-0033] Kooijman, M. N. , Kruithof, C. J. , Van Duijn, C. M. , Duijts, L. , Franco, O. H. , van, I. M. H. , De Jongste, J. C. , Klaver, C. C. , Van der Lugt, A. , Mackenbach, J. P. , Moll, H. A. , Peeters, R. P. , Raat, H. , Rings, E. H. , Rivadeneira, F. , Van der Schroeff, M. P. , Steegers, E. A. , Tiemeier, H. , Uitterlinden, A. G. , & Jaddoe, V. W. (2016). The generation R study: Design and cohort update 2017. Eur J Epidemiol, 31(12), 1243–1264. 10.1007/s10654-016-0224-9 28070760 PMC5233749

[jcv212278-bib-0034] Lyall, K. , Hosseini, M. , Ladd‐Acosta, C. , Ning, X. , Catellier, D. , Constantino, J. N. , Croen, L. A. , Kaat, A. J. , Botteron, K. , Bush, N. R. , Dager, S. R. , Duarte, C. S. , Fallin, M. D. , Hazlett, H. , Hertz‐Picciotto, I. , Joseph, R. M. , Karagas, M. R. , Korrick, S. , Landa, R. , program collaborators for Environmental influences on Child Health, O . (2021). Distributional properties and criterion validity of a shortened version of the social responsiveness scale: Results from the ECHO Program and implications for social communication research. J Autism Dev Disord, 51(7), 2241–2253. 10.1007/s10803-020-04667-1 32944847 PMC7965796

[jcv212278-bib-0035] MacKay, D. F. , Smith, G. C. , Dobbie, R. , & Pell, J. P. (2010). Gestational age at delivery and special educational need: Retrospective cohort study of 407,503 schoolchildren. PLoS Med, 7(6), e1000289. 10.1371/journal.pmed.1000289 20543995 PMC2882432

[jcv212278-bib-0036] Monk, C. , Lugo‐Candelas, C. , & Trumpff, C. (2019). Prenatal developmental origins of future psychopathology: Mechanisms and pathways. Annu Rev Clin Psychol, 15(1), 317–344. 10.1146/annurev-clinpsy-050718-095539 30795695 PMC7027196

[jcv212278-bib-0037] Ong, K. K. (2007). Catch‐up growth in small for gestational age babies: Good or bad? Curr Opin Endocrinol Diabetes Obes, 14(1), 30–34. 10.1097/MED.0b013e328013da6c 17940416

[jcv212278-bib-0038] Persson, M. , Opdahl, S. , Risnes, K. , Gross, R. , Kajantie, E. , Reichenberg, A. , Gissler, M. , & Sandin, S. (2020). Gestational age and the risk of autism spectrum disorder in Sweden, Finland, and Norway: A cohort study. PLoS Med, 17(9), e1003207. 10.1371/journal.pmed.1003207PMEDICINE-D-20-00561 32960896 PMC7508401

[jcv212278-bib-0039] Pettersson, E. , Larsson, H. , D'Onofrio, B. , Almqvist, C. , & Lichtenstein, P. (2019). Association of fetal growth with general and specific mental health conditions. JAMA Psychiatry, 76(5), 536–543. 10.1001/jamapsychiatry.2018.4342 30725083 PMC6495458

[jcv212278-bib-0040] Raven, J. (2021). The Raven’s 2 Progressive Matrices tests and manual. Pearson.

[jcv212278-bib-0041] Sacchi, C. , Marino, C. , Nosarti, C. , Vieno, A. , Visentin, S. , & Simonelli, A. (2020). Association of intrauterine growth restriction and small for gestational age status with childhood cognitive outcomes: A systematic review and meta‐analysis. JAMA Pediatr, 174(8), 772–781. 10.1001/jamapediatrics.2020.1097 32453414 PMC7251506

[jcv212278-bib-0042] Silva, C. C. V. M. , El Marroun, H. P. S. , S MD, PhD; Vernooij, M. W. MD, PhD; Muetzel, R. L. PhD;, & Susana Santos, P. V. W. V. J. , MD, PhD. (, Santos, S. , Jaddoe, V. W. V. 2021). Patterns of fetal and infant growth and brain morphology at age 10 years. JAMA Pediatr, *Vol.* 4(No. 12). e2138214, 10.1001/jamanetworkopen.2021.38214 PMC8662367 34882181

[jcv212278-bib-0043] Silveira, P. P. , Pokhvisneva, I. , Gaudreau, H. , Rifkin‐Graboi, A. , Broekman, B. F. P. , Steiner, M. , Levitan, R. , Parent, C. , Diorio, J. , & Meaney, M. J. (2018). Birth weight and catch up growth are associated with childhood impulsivity in two independent cohorts. Sci Rep, 8(1), 13705. 10.1038/s41598-018-31816-5 30209275 PMC6135839

[jcv212278-bib-0044] Song, I. G. , Kim, H. S. , Cho, Y. M. , Lim, Y. N. , Moon, D. S. , Shin, S. H. , Kim, E. K. , Park, J. , Shin, J. E. , Han, J. , & Eun, H. S. (2022). Association between birth weight and neurodevelopmental disorders assessed using the Korean National Health Insurance Service claims data. Sci Rep, 12(1), 2080. 10.1038/s41598-022-06094-x 35136157 PMC8827104

[jcv212278-bib-0045] Sterne, J. A. , White, I. R. , Carlin, J. B. , Spratt, M. , Royston, P. , Kenward, M. G. , Wood, A. M. , & Carpenter, J. R. (2009). Multiple imputation for missing data in epidemiological and clinical research: Potential and pitfalls. BMJ, 338(jun29 1), b2393. 10.1136/bmj.b2393 19564179 PMC2714692

[jcv212278-bib-0046] Talmi, Z. , Mankuta, D. , & Raz, R. (2020). Birth weight and autism spectrum disorder: A population‐based nested case‐control study. Autism Res, 13(4), 655–665. 10.1002/aur.2260 31930777

[jcv212278-bib-0047] Vandenbroucke, J. P. , Von Elm, E. , Altman, D. G. , Gotzsche, P. C. , Mulrow, C. D. , Pocock, S. J. , Poole, C. , Schlesselman, J. J. , Egger, M. , & Initiative, S. (2007). Strengthening the reporting of observational studies in Epidemiology (STROBE): Explanation and elaboration. PLoS Med, 4(10), e297. 10.1371/journal.pmed.0040297 17941715 PMC2020496

[jcv212278-bib-0048] Van Mil, N. H. , Steegers‐Theunissen, R. P. , Motazedi, E. , Jansen, P. W. , Jaddoe, V. W. , Steegers, E. A. , Verhulst, F. C. , & Tiemeier, H. (2015). Low and high birth weight and the risk of child attention problems. J Pediatr, 166(4), 862–869. 10.1016/j.jpeds.2014.12.075 25681197

[jcv212278-bib-0049] Velders, F. P. , Dieleman, G. , Henrichs, J. , Jaddoe, V. W. , Hofman, A. , Verhulst, F. C. , Hudziak, J. J. , & Tiemeier, H. (2011). Prenatal and postnatal psychological symptoms of parents and family functioning: The impact on child emotional and behavioural problems. Eur Child Adolesc Psychiatry, 20(7), 341–350. 10.1007/s00787-011-0178-0 21523465 PMC3135831

[jcv212278-bib-0050] Verburg, B. O. , Steegers, E. A. , De Ridder, M. , Snijders, R. J. , Smith, E. , Hofman, A. , Moll, H. A. , Jaddoe, V. W. , & Witteman, J. C. (2008). New charts for ultrasound dating of pregnancy and assessment of fetal growth: Longitudinal data from a population‐based cohort study. Ultrasound Obstet Gynecol, 31(4), 388–396. 10.1002/uog.5225 18348183

[jcv212278-bib-0051] Wagner, R. E. , Zhang, Y. , Gray, T. , Abbacchi, A. , Cormier, D. , Todorov, A. , & Constantino, J. N. (2019). Autism‐related variation in reciprocal social behavior: A longitudinal study. Child Dev, 90(2), 441–451. 10.1111/cdev.13170 30346626 PMC6446804

[jcv212278-bib-0052] Wolke, D. , Strauss, V. Y. , Johnson, S. , Gilmore, C. , Marlow, N. , & Jaekel, J. (2015). Universal gestational age effects on cognitive and basic mathematic processing: 2 cohorts in 2 countries. J Pediatr, 166(6), 1410–1416. e1411‐1412. 10.1016/j.jpeds.2015.02.065 25842966 PMC4441098

[jcv212278-bib-0053] Yang, S. , Tilling, K. , Martin, R. , Davies, N. , Ben‐Shlomo, Y. , & Kramer, M. S. (2011). Pre‐natal and post‐natal growth trajectories and childhood cognitive ability and mental health. Int J Epidemiol, 40(5), 1215–1226. 10.1093/ije/dyr094 21764769

