# Supplementary material for: Associations of fetal and infant growth patterns with behavior and cognitive outcomes in early adolescence
Source: JCPP Adv. 2024 Oct 23;5(3):e12278. doi: 10.1002/jcv2.12278 (PMC12446733; doi:10.1002/jcv2.12278)
Supplement: Supplementary file 1 — Supporting Information S1 [file JCV2-5-e12278-s001.docx]

**Original article**

**Associations of Fetal and Infant Growth Patterns with Behavior and Cognitive Outcomes in Early Adolescence**

*Short title: Early-life Growth and Behavior and Cognitive development*

**Supplementary Online Content**

**Supplementary Methods section**

**Supplementary Table S1. Observed participant characteristics of the study population**

**Supplementary Table S2. Non-response analysis in singleton live births with and without outcome measurements**

**Supplementary Table S3. Associations of birth outcomes with total, internalizing and externalizing problems in early adolescence, basic model**

**Supplementary Table S4. Associations of birth outcomes with ADHD symptoms, autism traits and IQ in early adolescence, basic model**

**Supplementary Table S5. Associations of fetal and infant growth patterns with total, internalizing and externalizing problems in early adolescence, basic model**

**Supplementary Table S6. Associations of fetal and infant growth patterns with ADHD symptoms, autism traits and IQ in early adolescence, basic model**

**Supplementary Table S7. Associations of birth and infant head circumference patterns with total, internalizing and externalizing problems in early adolescence**

**Supplementary Table S8. Associations of birth and infant head circumference patterns with ADHD symptoms, autism traits and IQ in early adolescence**

**Supplementary Table S9. Sensitivity analysis associations of fetal and infant growth patterns with total problem, internalizing, externalizing scores in early adolescence**

**Supplementary Table S10. Sensitivity analysis associations of fetal and infant growth patterns with ADHD symptoms, autism traits and IQ in early adolescence**

**Supplementary Table S11. Distribution of fetal and infant growth patterns in children with different birth characteristics**

**Supplementary Figure S1. Flowchart of participants**

**Supplementary Figure S2.**  **Directed acyclic graphs (DAG)**

**Supplementary Figure S3. Scatterplots of birth weight with behavior and cognitive outcomes**

**Supplementary Figure S4. Scatterplots of gestational age at birth with behavior and cognitive outcomes**

**Supplementary Figure S5. Scatterplots of gestational age and sex-adjusted birth weight with behavior and cognitive outcomes**

**Supplementary STROBE Checklist**

This supplementary material has been provided by the authors to give readers additional information about their work.

**Supplementary Methods section**

**Study population**

This study was embedded in the Generation R Study, a population-based prospective cohort study from early fetal life onward (Kooijman et al., 2016). Pregnant women with a delivery date between April 2002 and January 2006, living in Rotterdam, the Netherlands, were eligible for participation. We had information on fetal or infant growth in 8,624 singleton births. Included in the data collection were questionnaires, physical and ultrasound examinations, biological samples and behavioral observations, which were performed in mothers, fathers and children.

**Emotional, behavioral and cognitive outcomes**

At the 13-year-follow-up, the primary caregiver also completed an adapted version of the Social Responsiveness Scale, consisting of 18 items. This abbreviated version shows high correlations with the full scale (Lyall et al., 2021; Sturm et al., 2017). The SRS is a quantitative measure of autistic traits for children aged between 4 and 18 years (Cheon et al., 2016; Lyall et al., 2021; Wagner et al., 2019). The SRS aims to identify a wide spectrum of reciprocal social behavior deficits. Scoring is on a four-point Likert scale. The shortened 18-item SRS contained items from three subscales: social cognition, social communication and autistic mannerisms. The Crohnbach’s alpha indicated high inter-item reliability for the SRS (α = 0.92). The SRS allows the exploration of autism-related traits as a continuous measure with higher scores indicating greater social behavior impairment.

**REFERENCES**

Cheon, K. A., Park, J. I., Koh, Y. J., Song, J., Hong, H. J., Kim, Y. K., Lim, E. C., Kwon, H., Ha, M., Lim, M. H., Paik, K. C., Constantino, J. N., Leventhal, B., & Kim, Y. S. (2016). The social responsiveness scale in relation to DSM IV and DSM5 ASD in Korean children. *Autism Res*, *9*(9), 970-980. <https://doi.org/10.1002/aur.1671>

Kooijman, M. N., Kruithof, C. J., van Duijn, C. M., Duijts, L., Franco, O. H., van, I. M. H., de Jongste, J. C., Klaver, C. C., van der Lugt, A., Mackenbach, J. P., Moll, H. A., Peeters, R. P., Raat, H., Rings, E. H., Rivadeneira, F., van der Schroeff, M. P., Steegers, E. A., Tiemeier, H., Uitterlinden, A. G., . . . Jaddoe, V. W. (2016). The Generation R Study: design and cohort update 2017. *Eur J Epidemiol*, *31*(12), 1243-1264. <https://doi.org/10.1007/s10654-016-0224-9> 10.1007/s10654-016-0224-9 [pii]

Lyall, K., Hosseini, M., Ladd-Acosta, C., Ning, X., Catellier, D., Constantino, J. N., Croen, L. A., Kaat, A. J., Botteron, K., Bush, N. R., Dager, S. R., Duarte, C. S., Fallin, M. D., Hazlett, H., Hertz-Picciotto, I., Joseph, R. M., Karagas, M. R., Korrick, S., Landa, R., . . . program collaborators for Environmental influences on Child Health, O. (2021). Distributional Properties and Criterion Validity of a Shortened Version of the Social Responsiveness Scale: Results from the ECHO Program and Implications for Social Communication Research. *J Autism Dev Disord*, *51*(7), 2241-2253. <https://doi.org/10.1007/s10803-020-04667-1> 10.1007/s10803-020-04667-1 [pii]

Sturm, A., Kuhfeld, M., Kasari, C., & McCracken, J. T. (2017). Development and validation of an item response theory-based Social Responsiveness Scale short form. *J Child Psychol Psychiatry*, *58*(9), 1053-1061. <https://doi.org/10.1111/jcpp.12731>

Vandenbroucke, J. P., von Elm, E., Altman, D. G., Gotzsche, P. C., Mulrow, C. D., Pocock, S. J., Poole, C., Schlesselman, J. J., Egger, M., & Initiative, S. (2007). Strengthening the Reporting of Observational Studies in Epidemiology (STROBE): explanation and elaboration. *PLoS Med*, *4*(10), e297. <https://doi.org/07-PLME-RA-1056> [pii] 10.1371/journal.pmed.0040297

Wagner, R. E., Zhang, Y., Gray, T., Abbacchi, A., Cormier, D., Todorov, A., & Constantino, J. N. (2019). Autism-Related Variation in Reciprocal Social Behavior: A Longitudinal Study. *Child Dev*, *90*(2), 441-451. <https://doi.org/10.1111/cdev.13170>

**Supplementary TableS1. Descriptive statistics of the study population^a^**

| Characteristic | N. available cases | (N= 4716)  N (%) |
| --- | --- | --- |
| Maternal |  |  |
| Age at enrolment, median (95% range), years | 4716 | 31.4 (20.2, 39.6) |
| Pre-pregnancy BMI, median (95% range), kg/m^2^ | 3948 | 22.5 (18.2, 34.3) |
| Parity nulliparous, No. (%) | 4695 | 2745 (58.5) |
| Education level, No. (%) | 4488 |  |
| Primary education | 322 | 322 (7.2) |
| Secondary education | 1820 | 1820 (40.6) |
| Higher education | 2346 | 2346 (52.3) |
| Ethnicity, No. (%) | 4636 |  |
| Dutch | 2723 | 2723 (58.7) |
| Non-Dutch, Western | 385 | 385 (8.3) |
| Non-Dutch, Non-Western | 1528 | 1528 (33.0) |
| Smoking during pregnancy, No. (%) | 4235 | 984 (23.2) |
| Alcohol during pregnancy, No. (%) | 4195 | 2486 (59.3) |
| Folic acid supplement use, did not use No. (%) | 3660 | 747 (20.4) |
| Maternal IQ, mean (SD) | 2975 | 101.8 (17.4) |
| Fetal |  |  |
| Second trimester |  |  |
| Gestational age, median (95% range), weeks | 4510 | 20.5 (18.6, 23.3) |
| Estimated fetal weight, median (95% range), grams | 993 | 362 (260, 534) |
| Third trimester |  |  |
| Gestational age, median (95% range), weeks | 4605 | 30.4 (28.5, 32.9) |
| Estimated fetal weight, median (95% range), grams | 1007 | 1602 (1211, 2214) |
| Birth |  |  |
| Child sex, female No. (%) | 4716 | 2400 (50.9) |
| Gestational age at birth, median (95% range), weeks | 4716 | 40.1 (35.9, 42.4) |
| < 37 weeks, No. (%) | 216 | 216 (4.6) |
| 37,42 weeks, No. (%) | 4162 | 4162 (88.3) |
| >42 weeks, No. (%) | 338 | 338 (7.2) |
| Birth weight, median (95% range), grams | 4710 | 3470 (2254, 4500) |
| < 2500 grams, No. (%) | 201 | 201 (4.3) |
| 25004500 grams, No. (%) | 4388 | 4388 (93.2) |
| > 4500 grams, No. (%) | 221 | 121 (2.6) |
| Sex, and gestational age adjusted birth weight |  |  |
| Small (<10^th^ percentile), No. (%) | 471 | 471 (10.0) |
| Appropriate (10^th^,90^th^ percentile), No. (%) | 3768 | 3768 (80.0) |
| Large (>90^th^ percentile), No. (%) | 470 | 471 (10.0) |
| Postconceptional age at head circumference measurement, median (95% range), weeks | 2700 | 41.1 (37.6, 52.9) |
| Head circumference, mean (SD), cm | 2700 | 35.2 (2.4) |
| Infant |  |  |
| At 6 months visit |  |  |
| Age at visit, median (95% range), months | 3629 | 6.2 (5.2, 8.3) |
| Weight, median (95% range), kg | 3610 | 7.8 (6.2, 9.7) |
| At 12 months visit |  |  |
| Age at visit, median (95% range), years | 3326 | 11.1 (10.1, 12.5) |
| Weight, median (95% range), kg | 3310 | 9.6 (7.6, 11.8) |
| Head circumference, mean (SD), cm | 2700 | 46.1 (1.4) |
| At 2 year visit |  |  |
| Age at visit, median (95% range), years | 3103 | 24.9 (23.4, 28.2) |
| Weight, median (95% range), kg | 3097 | 12.8 (10.3, 16.0) |
| Childhood |  |  |
| Age at follow,up, median (95% range), years | 4338 | 13.5 (13.1, 14.7) |
| CBCL total sum score, median (95% range) | 4133 | 14.0 (0.0, 63.0) |
| CBCL internalizing score, median (95% range) | 4136 | 4.0 (0.0, 21.4) |
| CBCL externalizing score, median (95% range) | 4125 | 2.0 (0.0, 18.0) |
| CBCL ADHD trait score, median (95% range) | 4117 | 2.0 (0.0, 10.0) |
| SRS Autism score, median (95% range) | 4130 | 4.0 (0.0, 16.0) |
| Intelligence Quotient, mean (SD) | 4292 | 101.6 (13.9) |

BMI: body mass index, mm: millimeter, kg: kilograms, CBCL: Child Behavioral Checklist. DSM: Diagnostic and Statistical Manual of Mental Disorders. ADHD: attention-deficit hyperactivity disorder. SRS: Social Responsiveness Scale. Values are mean (SD), median (95% range), or number (valid %). ^a:^ Characteristics are based on observed not imputed data

**Supplementary TableS2. Non-response analysis in singleton live births with and without outcome measurements.**

| Characteristics | Children included in the analysis  (n=4716) | Children not included in the analysis (n=3906)* | p-value |
| --- | --- | --- | --- |
| Maternal |  |  |  |
| Age at enrolment, median (95% range), years | 31.4 (20.2, 39.6) | 28.4 (18.5, 38.5) | <0.001 |
| Pre-pregnancy BMI, median (95% range), kg/m^2^ | 22.7 (17.8, 34.1) | 22.8 (17.7, 35.9) | <0.01 |
| Parity, n nulliparous (%) | 2757 (58.5) | 2046 (52.4) | <0.001 |
| Education level, n higher education (%) | 2398 (50.7) | 1047 (26.8) | <0.001 |
| Ethnicity, n European (% | 3136 (66.5) | 1702 (43.6) | <0.001 |
| Smoking during pregnancy, n ever in pregnancy (%) | 1108 (23.5) | 1250 (32.0) | <0.001 |
| Alcohol during pregnancy, n ever in pregnancy (%) | 2753 (58.4) | 1523 (39.0) | <0.001 |
| Folic acid supplement use, n did not use (%) | 1066 (22.6) | 1657 (42.4) | <0.001 |
| Maternal IQ, mean (SD) | 99.8 (18.0) | 95.5 (18.7) | <0.001 |
| Fetal |  |  |  |
| Second trimester |  |  |  |
| Gestational age, median (95% range), weeks | 20.5 (18.6, 23.3) | 20.5 (18.5, 23.8) | 0.46 |
| Estimated fetal weight, median (95% range), grams | 3612 (260, 534) | 359 (247, 533) | 0.37 |
| Third trimester |  |  |  |
| Gestational age, median (95% range), weeks | 30.4 (28.5, 32.9) | 30.3 (27.9, 33.1) | <0.01 |
| Estimated fetal weight, median (95% range), grams | 1602 (1211, 2214) | 1597 (1176, 2131) | 0.38 |
| Birth |  |  |  |
| Child sex, n female (%) | 2400 (50.9) | 1874 (48.0) | <0.01 |
| Gestational age at birth, median (95% range), weeks | 40.1 (35.9, 42.4) | 40.0 (35.3, 42.3) | <0.001 |
| < 37 weeks, n (%) | 216 (4.6) | 235 (6.0) | <0.01 |
| 37-42 weeks, n (%) | 4162 (88.3) | 3406 (87.4) | <0.01 |
| >42 weeks, n (%) | 338 (7.2) | 257 (6.6) | <0.01 |
| Birth weight, median (95% range), grams | 3470 (2254, 4500) | 3380 (2200, 4450) | <0.001 |
| < 2500 grams, n (%) | 201 (4.3) | 214 (5.5) | 0.013 |
| 2500-4500 grams, n (%) | 4388 (93.2) | 3549 (92.2) | 0.013 |
| > 4500 grams, n (%) | 121 (2.6) | 85 (2.2) | 0.013 |
| Sex, and gestational age adjusted birth weight |  |  |  |
| Small (<10^th^ percentile), n (%) | 471 (10.0) | 463 (12.1) | <0.01 |
| Appropriate (10^th^,90^th^ percentile), n (%) | 3768 (80.0) | 3045 (79.4) | <0.01 |
| Large (>90^th^ percentile), n (%) | 471 (10.0) | 328 (8.6) | <0.01 |
| Postconceptional age at head circumference measurement, median (95% range), weeks | 41.1 (37.6, 52.9) | 40.7 (37.1, 52.3) | <0.001 |
| Head circumference, mean (SD), cm | 35.2 (2.4) | 34.8 (2.3) | <0.001 |
| Infant |  |  |  |
| At 6 months visit |  |  |  |
| Age at visit, median (95% range), months | 6.2 (5.2, 8.3) | 6.2 (5.2, 8.5) | 0.52 |
| Weight, median (95% range), kg | 7.8 (6.2, 9.7) | 7.9 (6.3, 10.0) | <0.001 |
| At 12 months visit |  |  |  |
| Age at visit, median (95% range), years | 11.1 (10.1, 12.5) | 11.1 (10.1, 12.6) | 0.68 |
| Weight, median (95% range), kg | 9.6 (7.6, 11.8) | 9.7 (7.7, 12.2) | <0.001 |
| Head circumference, mean (SD), cm | 46.1 (1.4) | 46.1 (1.5) | 0.27 |
| At 2 year visit |  |  |  |
| Age at visit, median (95% range), years | 24.9 (23.4, 28.2) | 24.7 (23.4, 28.3) | 0.28 |
| Weight, median (95% range), kg | 12.8 (10.3, 16.0) | 12.9 (10.1, 16.5) | 0.06 |

BMI: body mass index, mm: millimeter, kg: kilograms. * Children for whom no data on behavior or cognition was available. Values are mean (SD), median (95% range), or number (valid %). Differences in subject characteristics between the groups were evaluated using Independent Student T,test and Mann,Whitney U for continuous variables and χ^2^ tests for categorical variables

**Supplementary Table S3. Associations of birth outcomes with total, internalizing and externalizing behavior problems in early adolescence, basic model**

|  |  | Total problems  (n=4133) | Total problems  >80th percentile  (n=845)^a^ | Internalizing  problems  (n=4136) | Internalizing  problems  >80th percentile  (n= 932)^b^ | Externalizing  problems  (n=4125) | Externalizing  problems  >80th percentile (n=972)^c^ |
| --- | --- | --- | --- | --- | --- | --- | --- |
| Birth characteristics | N | Difference in SDS (95 CI) | OR (95% CI) | Difference in SDS (95 CI) | OR (95% CI) | Difference in SDS (95 CI) | OR (95% CI) |
| Gestational age at birth, wk |  | -0.03 (-0.05 to -0.01)* | 0.95 (0.91 to 0.99)* | -0.02 (-0.04 to -0.00)* | 0.97 (0.93 to 1.02) | -0.02 (-0.04 to 0.00)* | 0.95 (0.91 to 0.99)** |
| < 37 | 216 | 0.23 (0.08 to 0.38)* | 1.25 (0.88 to 1.77) | 0.17 (0.02 to 0.32)* | 1.21 (0.86 to 1.70) | 0.13 (-0.03 to 0.27) | 1.24 (0.88 to 1.73) |
| 37-42 | *4162* | *Reference* | *Reference* | *Reference* | *Reference* | *Reference* | *Reference* |
| >42 | 338 | 0.04 (-0.8 to 0.16) | 0.95 (0.71 to 1.28) | 0.02 (-0.10 to 0.14) | 0.99 (0.74 to 1.32) | 0.03 (-0.09 to 0.15) | 1.01 (0.76 to 1.33) |
| Birth weight, 500 gr |  | -0.04 (-0.07 to -0.02)* | 0.90 (0.85 to 0.97)** | -0.04 (-0.07 to -0.01)* | 0.91 (0.85 to 0.97)** | -0.03 (-0.06 to 0.00) | 0.91 (0.86 to 0.98)** |
| < 2500 | 201 | 0.17 (0.02 to 0.32)* | 1.20 (0.84 to 1.72) | 0.08 (-0.07 to 0.23) | 1.11 (0.78 to 1.57) | 0.12 (-0.03 to 0.28) | 1.28 (0.91 to 1.79) |
| 2500-4500 | *4388* | *Reference* | *Reference* | *Reference* | *Reference* | *Reference* | *Reference* |
| > 4500 | 121 | 0.07 (-0.12 to 0.27) | 0.90 (0.55 to 1.48) | -0.01 (-0.20 to 0.19) | 0.96 (0.60 to 1.55) | 0.12 (-0.07 to 0.32) | 0.88 (0.55 to 1.41) |
| Size for gestational age at birth, SD score |  | -0.02 (-0.05 to 0.00) | 0.93 (0.86 to 1.00) | -0.03 (-0.06 to 0.00) | 0.91 (0.84 to 0.98)** | -0.01 (-0.04 to 0.02) | 0.94 (0.88 to 1.01) |
| Small <10^th^ percentile | 471 | 0.16 (0.06 to 0.26)* | 1.42 (1.13 to 1.79)** | 0.18 (0.08 to 0.29)** | 1.66 (1.33 to 2.07)** | 0.10 (-0.01 to 0.20) | 1.23 (0.86 to 1.55) |
| Appropriate 10^th^-90^th^ percentile | *3768* | *Reference* | *Reference* | *Reference* | *Reference* | *Reference* | *Reference* |
| Large >90^th^ percentile | 470 | 0.02 (-0.09 to 0.12) | 1.00 (0.78 to 1.28) | -0.02 (-0.12 to 0.08) | 0.86 (0.66 to 1.11) | 0.03 (-0.07 to 0.14) | 1.01 (0.79 to 1.29) |

CI: Confidence interval, Wk: weeks, SDS: standard deviation score, OR: Odds Ratio. **P* value <0.05 ***P* value <0.01. Values are regression coefficients (95% confidence interval) or odds ratio’s (95% confidence interval) obtained from multivariable linear respectively logistic regression models and reflect the differences in parent-reported behavioral problems, the total problems (SDS), internalizing problems (SDS) and externalizing problems (SDS) for birth characteristics. For the logistic regressions the top 20% of the total score, the internalizing and the externalizing behavior problems score was used. Total number of available cases; ^a^: n=4133, ^b^: n=4136, ^c^: n=4125. Pooled estimates are from multiple imputed datasets. The basic model is adjusted for age at time of measurement and sex of the child.

**Supplementary TableS4. Associations of birth outcomes with ADHD symptoms, autism traits and IQ in early adolescence, basic model**

|  |  | ADHD symptoms score (n= 4117) | ADHD symptoms score  >80th percentile  (n=1009)^a^ | Autistic traits score  (n = 4130) | Autistic traits score  Cut-off  (n=362)^b^ | IQ score  (n = 4292) | IQ score  <80  (n =261)^c^ |
| --- | --- | --- | --- | --- | --- | --- | --- |
| Birth characteristics | N | Difference in SDS (95 CI) | OR (95% CI) | Difference in SDS (95 CI) | OR (95% CI) | Difference in SDS (95 CI) | OR (95% CI) |
| Gestational age at birth, wk |  | -0.03 (-0.05 to -0.01)* | 0.95 (0.91 to 0.99)* | -0.02 (-0.04 to -0.01)* | 0.94 (0.89 to 0.99)* | 0.03 (0.01 to 0.05)** | 0.89 (0.84 to 0.94)** |
| < 37 | 216 | 0.19 (0.43 to 0.34)* | 1.35 (0.97 to 1.88) | 0.06 (-0.09 to 0.21) | 1.21 (0.74 to 2.00) | -0.07 (-0.22 to 0.07) | 1.7 (1.05 to 2.78)* |
| 37-42 | *4162* | *Reference* | *Reference* | *Reference* | *Reference* | *Reference* | *Reference* |
| >42 | 338 | -0.01 (-0.13 to 0.11) | 0.97 (0.74 to 1.28) | -0.14 (-0.26 to -0.02)* | 0.92 (0.60 to 1.41) | 0.15 (0.04 to 0.27)* | 0.77 (0.45 to 1.32) |
| Birth weight, 500 gr |  | -0.04 (-0.07 to -0.01)* | 0.91 (0.86 to 0.98)** | -0.05 (-0.08 to -0.02)* | 0.89 (0.81 to 0.98)* | 0.09 (0.06 to 0.11)** | 0.76 (0.68 to 0.84)** |
| < 2500 | 201 | 0.19 (0.04 to 0.34)* | 1.35 (0.97 to 1.89) | 0.12 (-0.04 to 0.27) | 1.54 (0.97 to 2.44) | -0.08 (-0.23 to 0.06) | 1.78 (1.08 to 2.92)* |
| 2500-4500 | *4388* | *Reference* | *Reference* | *Reference* | *Reference* | *Reference* | *Reference* |
| > 4500 | 121 | 0.04 (-0.16 to 0.23) | 0.86 (0.54 to 1.36) | -0.04 (-0.24 to 0.16) | 0.68 (0.31 to 1.47) | 0.11 (-0.09 to 0.30) | 0.57 (0.21 to 1.58) |
| Size for gestational age at birth, SD score |  | -0.03 (-0.06 to 0.00) | 0.94 (0.88 to 1.01) | -0.04 (-0.07 to 0.01)* | 0.92 (0.83 to 1.02) | 0.09 (0.06 to 0.12)** | 0.79 (0.70 to 0.90)** |
| Small <10^th^ percentile | 471 | 0.15 (0.06 to 0.24)* | 1.23 (0.98 to 1.55) | 0.12 (0.02 to 0.22)* | 1.27 (0.91 to 1.78) | -0.20 (-0.30 to -0.10)** | 1.62 (1.13 to 2.33)** |
| Appropriate 10^th^-90^th^ percentile | *3768* | *Reference* | *Reference* | *Reference* | *Reference* | *Reference* | *Reference* |
| Large >90^th^ percentile | 470 | -0.00 (-0.10 to 0.10) | 0.98 (0.77 to 1.24) | -0.04 (-0.14 to 0.06) | 0.82 (0.55 to 1.21) | 0.15 (0.05 to 0.25)* | 0.96 (0.62 to 1.50) |

ADHD: attention-deficit hyperactivity disorder, IQ: Intelligence Quotient, SDS: standard deviation score, OR: Odds Ratio, CI: Confidence interval. **P* value <0.05 ***P* value <0.01. Values are regression coefficients (95% confidence interval) or odds ratio’s (95% confidence interval) obtained from multivariable linear respectively logistic regression models and reflect the differences in parent-reported behavioral problems, ADHD symptoms score (SDS), autistic traits score (SDS) and IQ (SDS) for birth characteristics. For the logistic regressions the top 20% of the ADHD symptoms score, autistic traits score (weighted scores of >1.078 for boys and >1.000 for girls) and IQ (score <79) was used. Total number of available cases; ^a^: n=4117, ^b^: n=4130, ^c^: n=4292. Pooled estimates are from multiple imputed datasets. The basic model is adjusted for age at time of measurement and sex of the child.

**Supplementary TableS5. Associations of fetal and infant growth patterns with total, internalizing and externalizing behavior problems in early adolescence, basic model**

|  |  | Total problems  (n= 3315) | Total problems  >80th percentile  (n=644)^a^ | Internalizing problems (n=3318) | Internalizing  problems >80th percentile (n=723)^b^ | Externalizing  problems (n=3309) | Externalizing problems >80th percentile  (n=747)^c^ |
| --- | --- | --- | --- | --- | --- | --- | --- |
| Fetal and infant growth patterns | N | Difference in SDS (95 CI) | OR (95% CI) | Difference in SDS (95 CI) | OR (95% CI) | Difference in SDS (95 CI) | OR (95% CI) |
| Fetal growth deceleration |  |  |  |  |  |  |  |
| Infant growth deceleration | 129 | 0.04 (-0.16 to 0.23) | 0.91 (0.57 to 1.48) | 0.07 (-0.12 to 0.27) | 0.85 (0.52 to 1.37) | -0.12 (-0.31 to 0.08) | 0.72 (0.44 to 1.18) |
| Infant normal growth | 408 | -0.04 (-0.17 to 0.08) | 0.69 (0.51 to 0.94)* | -0.00 (-0.12 to 0.12) | 0.81 (0.60 to 1.10) | -0.08 (-0.20 to 0.04) | 0.88 (0.66 to 1.18) |
| Infant growth acceleration | 419 | 0.01 (-0.11 to 0.14) | 0.88 (0.65 to 1.20) | 0.04 (-0.09 to 0.16) | 0.98 (0.73 to 1.32) | 0.03 (-0.10 to 0.15) | 0.99 (0.74 to 1.32) |
| Fetal normal growth |  |  |  |  |  |  |  |
| Infant growth deceleration | 351 | 0.02 (-0.11 to 0.15) | 0.95 (0.69 to 1.30) | 0.02 (-0.11 to 0.15) | 1.02 (0.75 to 1.38) | 0.02 (-0.11 to 0.15) | 1.03 (0.76 to 1.39) |
| Infant normal growth | *869* | *Reference* | *Reference* | *Reference* | *Reference* | *Reference* | *Reference* |
| Infant growth acceleration | 398 | -0.04 (-0.16 to 0.09) | 0.73 (0.53 to 1.01) | 0.01 (-0.12 to 0.14) | 0.89 (0.65 to 1.21) | -0.04 (-0.17 to 0.09) | 0.77 (0.56 to 1.05) |
| Fetal growth acceleration |  |  |  |  |  |  |  |
| Infant growth deceleration | 479 | 0.01 (-0.05 to 0.07) | 0.79 (0.63 to 1.00) | -0.03 (-0.15 to 0.09) | 0.84 (0.64 to 1.11) | -0.01 (-0.12 to 0.11) | 0.82 (0.62 to 1.09) |
| Infant normal growth | 505 | -0.14 (-0.25 to -0.02)* | 0.67 (0.51 to 0.88)** | -0.12 (-0.23 to -0.00)* | 0.79 (0.59 to 1.04) | -0.09 (-0.21 to 0.02) | 0.80 (0.61 to 1.06) |
| Infant growth acceleration | 150 | -0.08 (-0.26 to 0.11) | 0.86 (0.54 to 1.36) | -0.05 (-0.24 to 0.13) | 0.77 (0.49 to 1.22) | -0.01 (-0.19 to 0.17) | 0.80 (0.51 to 1.27) |

CI: Confidence interval, SDS: standard deviation score, OR: Odds Ratio. **P* value <0.05 ***P* value <0.01. Values are regression coefficients (95% confidence interval) or odds ratio’s (95% confidence interval) obtained from multivariable linear respectively logistic regression models and reflect the differences in parent-reported behavioral problems, the total problems (SDS), internalizing problems (SDS) and externalizing problems (SDS) for fetal and infant growth patterns. For the logistic regressions the top 20% of the total problems, the internalizing and the externalizing behavior problems was used. Total number of available cases; ^a^: n=3315, ^b^: n=3318, ^c^: n=3309. Pooled estimates are from multiple imputed datasets. The basic model is adjusted for age at time of measurement and sex of the child.

**Supplementary TableS6. Associations of fetal and infant growth patterns with ADHD symptoms, autism traits and IQ in early adolescence, basic model**

|  |  | ADHD symptoms score  (n= 3304) | ADHD symptoms score  >80th percentile (n=764)^a^ | Autistic traits score  (n =3315) | Autistic traits score  Cut-off  (n=274)^b^ | IQ score  (n = 3348) | IQ score  <80  (n =171)^c^ |
| --- | --- | --- | --- | --- | --- | --- | --- |
| Fetal and infant growth patterns | N | Difference in SDS (95 CI) | OR (95% CI) | Difference in SDS (95 CI) | OR (95% CI) | Difference in SDS (95 CI) | OR (95% CI) |
| Fetal growth deceleration |  |  |  |  |  |  |  |
| Infant growth deceleration | 129 | -0.06 (-0.25 to 0.14) | 0.92 (0.62 to 1.37) | -0.10 (-0.29 to 0.10) | 0.61 (0.27 to 1.37) | -0.04 (-0.23 to 0.15) | 1.72 (0.86 to 3.45) |
| Infant normal growth | 408 | 0.05 (-0.07 to 0.17) | 1.01 (0.76 to 3.35) | 0.01 (-0.11 to 0.13) | 0.85 (0.55 to 1.32) | -0.10 (-0.22 to 0.02) | 1.20 (0.72 to 2.00) |
| Infant growth acceleration | 419 | 0.01 (-0.11 to 0.13) | 1.01 (0.75 to 3.35) | -0.05 (-0.18 to 0.07) | 0.87 (0.56 to 1.36) | -0.03(-0.15 to 0.09) | 0.59 (0.31 to 1.11) |
| Fetal normal growth |  |  |  |  |  |  |  |
| Infant growth deceleration | 351 | 0.01 (-0.12 to 0.14) | 0.82 (0.59 to 1.13) | -0.01 (-0.14 to 0.12) | 1.05 (0.68 to 1.63) | 0.06 (-0.06 to 0.19) | 0.69 (0.37 to 1.30) |
| Infant normal growth | *869* | *Reference* | *Reference* | *Reference* | *Reference* | *Reference* | *Reference* |
| Infant growth acceleration | 398 | -0.03 (-0.16 to 0.10) | 0.86 (0.63 to 1.17) | -0.03 (-0.15 to 0.10) | 0.60 (0.36 to 0.99)* | 0.04 (-0.09 to 0.16) | 0.98 (0.57 to 1.69) |
| Fetal growth acceleration |  |  |  |  |  |  |  |
| Infant growth deceleration | 479 | 0.08 (-0.04 to 0.19) | 0.98 (0.74 to 1.28) | -0.09 (-0.21 to 0.03) | 0.61 (0.39 to 0.97)* | 0.11 (-0.01 to 0.22) | 0.81 (0.47 to 1.39) |
| Infant normal growth | 505 | -0.08 (-0.19 to 0.03) | 0.89 (0.67 to 1.17) | -0.10 (-0.21 to 0.02) | 0.92 (0.62 to 1.38) | 0.10 (-0.14 to 0.21) | 0.79 (0.46 to 1.35) |
| Infant growth acceleration | 150 | -0.12 (-0.31 to 0.06) | 0.83 (0.66 to 1.05) | -0.03 (-0.21 to 0.16) | 0.89 (0.46 to 1.74) | 0.18 (-0.00 to 0.36) | 0.63 (0.25 to 1.63) |

ADHD: attention-deficit hyperactivity disorder, IQ: Intelligence Quotient, SDS: standard deviation score, OR: Odds Ratio, CI: Confidence interval. **P* value <0.05 ***P* value <0.01. Values are regression coefficients (95% confidence interval) or odds ratio’s (95% confidence interval) obtained from multivariable linear respectively logistic regression models and reflect the differences in parent-reported behavioral problems, ADHD symptoms score (SDS), autistic traits score (SDS) and IQ (SDS) for fetal and infant growth patterns. For the logistic regressions the top 20% of the ADHD symptoms score, autistic traits score (weighted scores of >1.078 for boys and >1.000 for girls) and IQ (score <79) was used. Total number of available cases; ^a^: n=3304, ^b^: n=3315, ^c^: n=3348. Pooled estimates are from multiple imputed datasets. The basic model is adjusted for age at time of measurement and sex of the child.

**Supplementary TableS7. Associations of birth and infant head circumference patterns with total, internalizing and externalizing bhevaior problems in early adolescence**

|  |  | Total problems  (n=2434) | Total problems >80th percentile  (n=468)^a^ | Internalizing problems (n=2437) | Internalizing problems >80th percentile (n=520)^b^ | Externalizing  problems (n=2429) | Externalizing problems >80th percentile  (n=539)^c^ |
| --- | --- | --- | --- | --- | --- | --- | --- |
| Birth and infant head circumference patterns | N | Difference in SDS (95 CI) | OR (95% CI) | Difference in SDS (95 CI) | OR (95% CI) | Difference in SDS (95 CI) | OR (95% CI) |
| Birth smallest tertile |  |  |  |  |  |  |  |
| Infant smallest tertile | 183 | 0.26 (0.10 to 0.42)** | 1.38 (0.94 to 2.04) | 0.09 (-0.07 to 0.26) | 1.21 (0.82 to 1.78) | 0.24 (0.07 to 0.40)** | 1.67 (1.15 to 2.41)** |
| Infant middle tertile | 516 | 0.09 (-0.02 to 0.20) | 0.94 (0.71 to 1.25) | 0.02 (-0.09 to 0.13) | 0.95 (0.73 to 1.25) | 0.08 (-0.03 to 0.18) | 1.10 (0.84 to 1.43) |
| Infant largest tertile | 45 | 0.27 (-0.04 to 0.58) | 1.59 (0.79 to 3.20) | 0.28 (-0.03 to 0.59) | 1.84 (0.94 to 3.61) | 0.05 (-0.26 to 0.37) | 1.33 (0.65 to 2.73) |
| Birth middle tertile |  |  |  |  |  |  |  |
| Infant smallest tertile | 164 | 0.06 (-0.11 to 0.22) | 0.90 (0.58 to 1.41) | 0.03 (-0.13 to 0.20) | 1.19 (0.79 to 1.77) | -0.06 (-0.23 to 0.10) | 1.05 (0.70 to 1.60) |
| Infant middle tertile | *1287* | *Reference* | *Reference* | *Reference* | *Reference* | *Reference* | *Reference* |
| Infant largest tertile | 240 | -0.01 (-0.15 to 0.14) | 0.73 (0.48 to 1.09) | -0.06 (-0.21 to 0.08) | 0.82 (0.56 to 1.19) | 0.02 (-0.12 to 0.16) | 0.99 (0.69 to 1.42) |
| Birth largest tertile |  |  |  |  |  |  |  |
| Infant smallest tertile | 12 | 0.23 (-0.36 to 0.81) | 0.41 (0.05 to 3.24) | 0.02 (-0.57 to 0.61) | 0.83 (0.18 to 3.88) | 0.17 (-0.42 to 0.75) | -- |
| Infant middle tertile | 171 | 0.16 (-0.01 to 0.33) | 1.04 (0.67 to 1.59) | 0.04 (-0.13 to 0.21) | 1.11 (0.74 to 1.66) | 0.10 (-0.07 to 0.26) | 1.28 (0.86 to 1.90) |
| Infant largest tertile | 82 | -0.26 (-0.49 to -0.03)* | 0.64 (0.32 to 1.27) | -0.19 (-0.43 to 0.04) | 0.77 (0.41 to 1.43) | -0.26 (-0.50 to -0.04)* | 0.58 (0.29 to 1.15) |

CI: Confidence interval, SDS: standard deviation score, OR: Odds Ratio. **P* value <0.05 ***P* value <0.01. Values are regression coefficients (95% confidence interval) or odds ratio’s (95% confidence interval) obtained from multivariable linear respectively logistic regression models and reflect the differences in parent-reported behavioral problems, the total problems (SDS), internalizing problems (SDS) and externalizing problems (SDS) for birth and infant head circumference patterns. For the logistic regressions the top 20% of the total problems, the internalizing and the externalizing behavior problems was used. Total number of available cases; ^a^: n=2434, ^b^: n=2437, ^c^: n=2429. Pooled estimates are from multiple imputed datasets. The confounder model is adjusted for age at time of measurement and sex of the child, maternal age in pregnancy, parity, pre-pregnancy body mass index, educational level, ethnicity, folic acid use, smoking, alcohol and maternal IQ measured at the child age of six years old.

**Supplementary TableS8. Associations of birth and infant head circumference patterns with ADHD symptoms, autism traits and IQ in early adolescence**

|  |  | ADHD symptoms score  (n=2425) | ADHD symptoms score  >80th percentile (n=532)^a^ | Autistic traits score  (n=2435) | Autistic traits score  Cut-off  (n=195)^b^ | IQ score  (n=2433) | IQ score  <80  (n=119)^c^ |
| --- | --- | --- | --- | --- | --- | --- | --- |
| Birth and infant head circumference patterns | N | Difference in SDS (95 CI) | OR (95% CI) | Difference in SDS (95 CI) | OR (95% CI) | Difference in SDS (95 CI) | OR (95% CI) |
| Birth smallest tertile |  |  |  |  |  |  |  |
| Infant smallest tertile | 183 | 0.31 (0.15 to 0.47)** | 1.77 (1.22 to 2.57)** | 0.17 (0.01 to 0.34)* | 1.04 (0.56 to 1.92) | -0.31 (-0.46 to -0.16)** | 2.64 (1.48 to 4.70)** |
| Infant middle tertile | 516 | 0.10 (-0.001 to 0.21) | 1.21 (0.93 to 1.58) | 0.05 (-0.06 to 0.16) | 1.20 (0.81 to 1.77) | -0.09 (-0.19 to 0.01) | 1.13 (0.67 to 1.90) |
| Infant largest tertile | 45 | 0.28 (-0.03 to 0.59) | 1.24 (0.59 to 2.59) | -0.18 (-0.49 to 0.13) | 1.57 (0.60 to 4.14) | -0.02 (-0.29 to 0.26) | 1.40 (0.41 to 4.82) |
| Birth middle tertile |  |  |  |  |  |  |  |
| Infant smallest tertile | 164 | 0.13 (-0.04 to 0.29) | 1.22 (0.82 to 1.84) | 0.04 (-0.13 to 0.21) | 1.39 (0.79 to 2.44) | -0.09 (-0.25 to 0.07) | 0.63 (0.22 to 1.82) |
| Infant middle tertile | *1287* | *Reference* | *Reference* | *Reference* | *Reference* | *Reference* | *Reference* |
| Infant largest tertile | 240 | -0.02 (-0.16 to 0.13) | 0.83 (0.56 to 1.22) | -0.09 (-0.24 to 0.05) | 0.61 (0.31 to 1.19) | 0.14 (0.01 to 0.27)* | 0.57 (0.22 to 1.47) |
| Birth largest tertile |  |  |  |  |  |  |  |
| Infant smallest tertile | 12 | 0.28 (-0.30 to 0.85) | 0.41 (0.05 to 3.22) | -0.06 (-0.63 to 0.51) | 0.94 (0.12 to 7.45) | -0.23 (-0.75 to 0.30) | 1.54 (0.18 to 13.15) |
| Infant middle tertile | 171 | 0.22 (0.06 to 0.38)** | 1.36 (0.92 to 2.03) | 0.06 (0.10 to 0.23) | 1.05 (0.56 to 1.97) | -0.10 (-0.25 to 0.06) | 0.94 (0.39 to 2.29) |
| Infant largest tertile | 82 | -0.22 (-0.45 to 0.01) | 0.54 (0.26 to 1.10) | -0.08 (-0.32 to 0.15) | 0.88 (0.34 to 2.25) | -0.04 (-0.25 to 0.18) | 0.89 (0.27 to 3.0) |

ADHD: attention-deficit hyperactivity disorder, IQ: Intelligence Quotient, SDS: standard deviation score, OR: Odds Ratio, CI: Confidence interval. **P* value <0.05. ***P* value <0.01. Values are regression coefficients (95% confidence interval) or odds ratio’s (95% confidence interval) obtained from multivariable linear respectively logistic regression models and reflect the differences in parent-reported behavioral problems, ADHD symptoms score (SDS), autistic traits score (SDS) and IQ (SDS) for birth and infant head circumference patterns.. For the logistic regressions the top 20% of the ADHD symptoms score, autistic traits score (weighted scores of >1.078 for boys and >1.000 for girls) and IQ (score <79) was used. Total number of available cases; ^a^: n=2425, ^b^: n=2435, ^c^: n=2433. Pooled estimates are from multiple imputed datasets. The confounder model is adjusted for age at time of measurement and sex of the child, maternal age in pregnancy, parity, pre-pregnancy body mass index, educational level, ethnicity, folic acid use, smoking, alcohol and maternal IQ measured at the child age of six years old.

**Supplementary TableS9. Sensitivity analysis associations of fetal and infant growth patterns with total problem, internalizing, externalizing scores in early adolescence**

|  |  | Total problem score  (n=2668) | Total problem score  >80th percentile  (n=506)^a^ | Internalizing problem  score  (n=2670) | Internalizing  problem  score  >80th percentile (n=580)^b^ | Externalizing  problem  score  (n=2664) | Externalizing problem  score  >80th percentile  (n=590)^c^ |
| --- | --- | --- | --- | --- | --- | --- | --- |
| Fetal and infant growth patterns | N | Difference in SDS (95 CI) | OR (95% CI) | Difference in SDS (95 CI) | OR (95% CI) | Difference in SDS (95 CI) | OR (95% CI) |
| Fetal growth deceleration |  |  |  |  |  |  |  |
| Infant growth deceleration | 114 | 0.11 (-0.10 to 0.31) | 0.99 (0.59 to 1.67) | 0.12 (-0.09 to 0.32) | 0.88 (0.52 to 1.47) | -0.05 (-0.26 to 0.15) | 0.88 (0.52 to 1.48) |
| Infant normal growth | 320 | -0.03 (-0.17 to 0.11) | 0.74 (0.51 to 1.07) | 0.002 (-0.14 to 0.14) | 0.82 (0.58 to 1.16) | -0.05 (-0.19 to 0.08) | 0.93 (0.66 to 1.30) |
| Infant growth acceleration | 331 | 0.03 (-0.11 to 0.17) | 0.94 (0.66 to 1.34) | 0.06 (-0.08 to 0.20) | 1.02 (0.73 to 1.42) | 0.06 (-0.08 to 0.20) | 1.13 (0.81 to 1.58) |
| Fetal normal growth |  |  |  |  |  |  |  |
| Infant growth deceleration | 285 | 0.04 (-0.11 to 0.18) | 1.06 (0.74 to 1.52) | 0.04 (-0.11 to 0.18) | 1.08 (0.77 to 1.53) | 0.06 (-0.08 to 0.20) | 1.13 (0.80 to 1.59) |
| Infant normal growth | *695* | *Reference* | *Reference* | *Reference* | *Reference* | *Reference* | *Reference* |
| Infant growth acceleration | 297 | -0.01 (-0.15 to 0.13) | 0.88 (0.61 to 1.27) | 0.07 (-0.07 to 0.21) | 0.99 (0.70 to 1.40) | 0.01 (-0.13 to 0.15) | 0.94 (0.66 to 1.34) |
| Fetal growth acceleration |  |  |  |  |  |  |  |
| Infant growth deceleration | 373 | 0.01 (-0.12 to 0.14) | 0.78 (0.55 to 1.11) | -0.003 (-0.14 to 0.13) | 0.90 (0.65 to 1.25) | -0.01 (0.14 to 0.12) | 0.77 (0.55 to 1.07) |
| Infant normal growth | 410 | -0.12 (-0.24 to 0.01) | 0.71 (0.50 to 0.995)* | -0.10 (-0.23 to 0.03) | 0.86 (0.63 to 1.18) | -0.06 (-0.19 to 0.07) | 0.90 (0.65 to 1.23) |
| Infant growth acceleration | 124 | -0.08 (-0.28 to 0.12) | 0.91 (0.55 to 1.52) | -0.05 (-0.25 to 0.15) | 0.48 (0.48 to 1.32) | -0.03 (-0.23 to 0.17) | 0.77 (0.46 to 1.29) |
| CI: Confidence interval, SDS: standard deviation score, OR: Odds Ratio. **P* value <0.05. Sensitvity analysis including only children with weight at 24 months old. Values are regression coefficients (95% confidence interval) or odds ratio’s (95% confidence interval) obtained from multivariable linear respectively logistic regression models and reflect the differences in parent-reported behavioral problems, the total problems (SDS), internalizing problems (SDS) and externalizing problems (SDS) for fetal and infant growth patterns. For the logistic regressions the top 20% of the total problems, the internalizing and the externalizing behavior problems was used. Total number of available cases; ^a^: n=2668, ^b^: n=2670, ^c^: n=2664. Pooled estimates are from multiple imputed datasets. The confounder model is adjusted for age at time of measurement and sex of the child, maternal age in pregnancy, parity, pre-pregnancy body mass index, educational level, ethnicity, folic acid use, smoking, alcohol and maternal IQ measured at the child age of six years old. | | | | | | | |

**Supplementary TableS10. Sensitivity analysis associations of fetal and infant growth patterns with ADHD symptoms, autism traits and IQ in early adolescence**

|  |  | | | ADHD symptoms score  (n= 2660) | ADHD symptoms score  >80th percentile (n=610)^a^ | Autistic traits score  (n = 2669) | Autistic traits score  Cut-off  (n=221)^b^ | IQ score  (n = 2658) | IQ score  <80  (n =124)^c^ |
| --- | --- | --- | --- | --- | --- | --- | --- | --- | --- |
| Fetal and infant growth patterns | | | N | Difference in SDS (95 CI) | OR (95% CI) | Difference in SDS (95 CI) | OR (95% CI) | Difference in SDS (95 CI) | OR (95% CI) |
| Fetal growth deceleration | |  | |  |  |  |  |  |  |
| Infant growth deceleration | | 114 | | -0.01 (-0.21 to 0.20) | 1.01 (0.61 to 1.67) | -0.09 (-0.29 to 0.12) | 0.74 (0.33 to 1.71) | -0.05 (-0.25 to 0.14) | 2.21 (1.01 to 4.83)* |
| Infant normal growth | | 320 | | 0.06 (-0.08 to 0.19) | 0.99 (0.71 to 1.38) | 0.01 (-0.13 to 0.15) | 0.91 (0.55 to 1.52) | -0.10 (-0.23 to 0.03) | 1.10 (0.59 to 2.07) |
| Infant growth acceleration | | 331 | | 0.001 (-0.14 to 0.14) | 1.08 (0.78 to 1.51) | -0.12 (-0.26 to 0.02) | 0.95 (0.57 to 1.59) | 0.03 (-0.10 to 0.16) | 0.50 (0.23 to 1.08) |
| Fetal normal growth | |  | |  |  |  |  |  |  |
| Infant growth deceleration | | 285 | | 0.004 (-0.14 to 0.15) | 0.83 (0.58 to 1.19) | -0.02 (-0.16 to 0.13) | 1.19 (0.73 to 1.94) | 0.05 (-0.09 to 0.19) | 0.73 (0.35 to 1.56) |
| Infant normal growth | | *695* | | *Reference* | *Reference* | *Reference* | *Reference* | *Reference* | *Reference* |
| Infant growth acceleration | | 297 | | -0.03 (-0.17 to 0.11) | 0.87 (0.61 to 1.24) | -0.05 (-0.19 to 0.09) | 0.81 (0.47 to 1.40) | 0.06 (-0.07 to 0.19) | 1.00 (0.52 to 1.92) |
| Fetal growth acceleration | |  | |  |  |  |  |  |  |
| Infant growth deceleration | | 373 | | 0.05 (-0.09 to 0.18) | 0.94 (0.68 to 1.29) | -0.09 (-0.23 to 0.04) | 0.64 (0.37 to 1.09) | 0.06 (-0.06 to 0.19) | 0.99 (0.51 to 1.91) |
| Infant normal growth | | 410 | | -0.07 (-0.20 to 0.05) | 0.89 (0.65 to 1.22) | -0.11 (-0.24 to 0.02) | 1.09 (0.69 to 1.72) | 0.06 (-0.06 to 0.18) | 0.78 (0.40 to 1.52) |
| Infant growth acceleration | | 124 | | -0.16 (-0.35 to 0.04) | 0.80 (0.48 to 1.33) | -0.04 (-0.24 to 0.16) | 0.99 (0.48 to 2.02) | 0.24 (0.05 to 0.43)* | 0.43 (0.13 to 1.48) |
| ADHD: attention-deficit hyperactivity disorder, IQ: Intelligence Quotient, SDS: standard deviation score, OR: Odds Ratio, CI: Confidence interval. **P* value <0.05. Sensitvity analysis including only children with weight at 24 months old. Values are regression coefficients (95% confidence interval) or odds ratio’s (95% confidence interval) obtained from multivariable linear respectively logistic regression models and reflect the differences in parent-reported behavioral problems, ADHD symptoms score (SDS), autistic traits score (SDS) and IQ (SDS) for fetal and infant growth patterns. For the logistic regressions the top 20% of the ADHD symptoms score, autistic traits score (weighted scores of >1.078 for boys and >1.000 for girls) and IQ (score <80) was used. Total number of available cases; ^a^: n=2660, ^b^: n=2669, ^c^: n=2658. Pooled estimates are from multiple imputed datasets. The confounder model is adjusted for age at time of measurement and sex of the child, maternal age in pregnancy, parity, pre-pregnancy body mass index, educational level, ethnicity, folic acid use, smoking, alcohol and maternal IQ measured at the child age of six years old. | | | | | | | | | |

**Supplementary Table S11. Distribution of fetal and infant growth patterns in children with different birth characteristics**

|  | Complete group  (n=3340) | Preterm born  <37 weeks  (n=150) | Low birth weight  <2500 grams  (n=135) | Small size for gestational age  <10^th^ percentile  (n=260) |
| --- | --- | --- | --- | --- |
| Fetal and infant growth patterns |  |  |  |  |
| Fetal growth deceleration | N | N | N | N |
| Infant growth deceleration | 129 | 16 | 9 | 4 |
| Infant normal growth | 408 | 23 | 26 | 43 |
| Infant growth acceleration | 419 | 12 | 47 | 130 |
| Fetal normal growth |  |  |  |  |
| Infant growth deceleration | 351 | 19 | 8 | 4 |
| Infant normal growth | *869* | *34* | *19* | *30* |
| Infant growth acceleration | 398 | 17 | 14 | 48 |
| Fetal growth acceleration |  |  |  |  |
| Infant growth deceleration | 479 | 15 | 5 | 0 |
| Infant normal growth | 505 | 13 | 6 | 0 |
| Infant growth acceleration | 150 | 1 | 1 | 1 |
| Number of participants per population, complete group of participants, children born preterm (<37 weeks of gestation), children born low birth weight (<2500 grams) and children born small size for gestational age (<10^th^ percentile of our study population). | | | | |

**Supplementary Figures**

**Supplementary Figure 1.** Flow chart of the study population

**n= 4716
Population for analyses:
Children with at least two consecutive weight measurements and attention problem score and/or IQ measurement and/or Social responsiveness Scale score and/or**

Total problem score, n= 4133

Internalizing score, n= 4136

Externalizing score, n= 4125

Attention-deficit hyperactivity disorder traits score, n= 4117

Autism disorder traits score, n= 4130

IQ measurement, n= 4292

**n= 3907 excluded:**Children without Child Behavior Checklist, n = 583

Children without IQ measurement, n = 424

Children without Social Responsiveness Scale score, n= 586

Ch

s

**n= 8623**

Singleton live births with data on sex and fetal or infant growth available

**n= 8632**Singleton live births

**n= 9 excluded:**Children with no data on sex and fetal, birth or infant growth measurements

**n= 246 excluded:**

Twin pregnancies, n= 97, APLA n=29

IUVD n= 75, Loss to follow-up n= 45

**n= 8878**Mothers enrolled in pregnancy in the Generation R Study

**
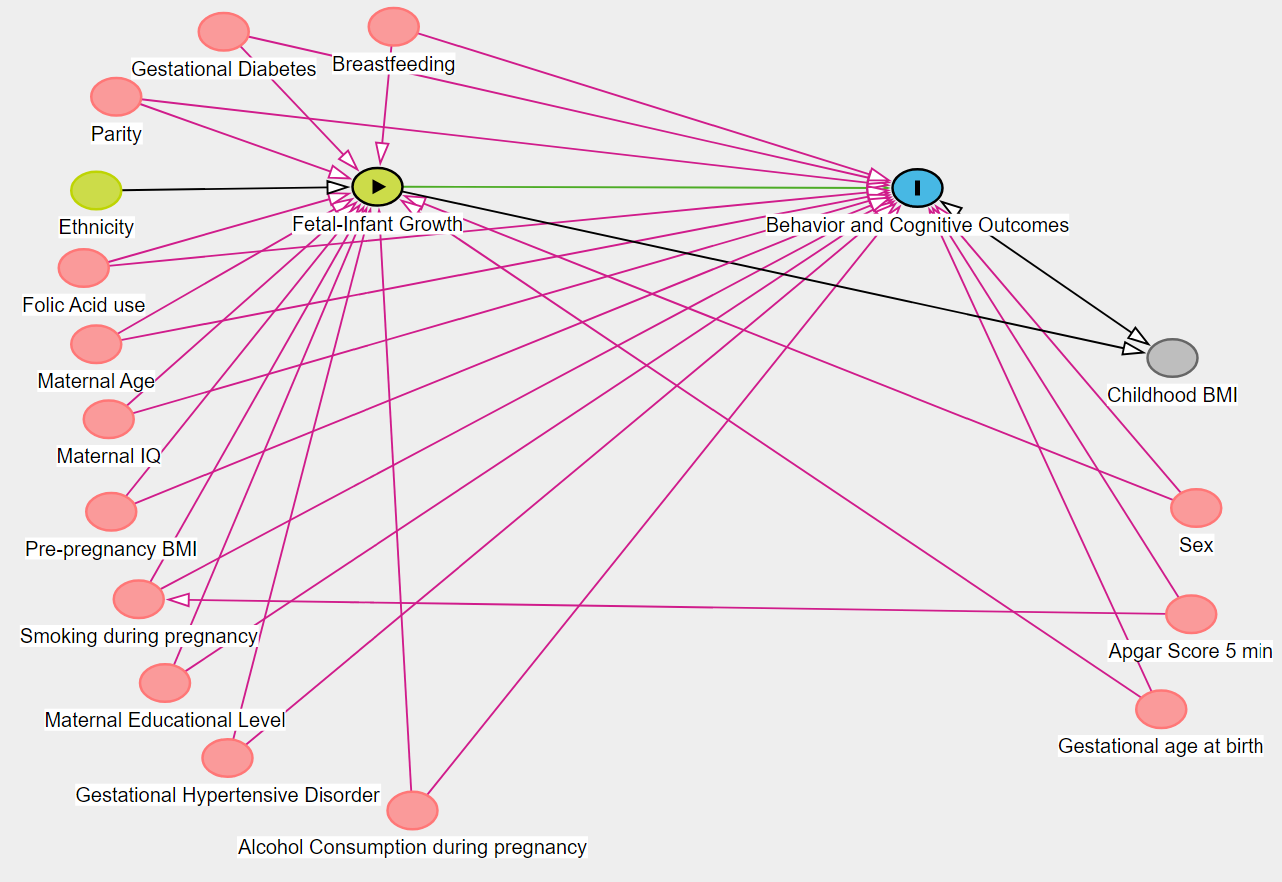
**

**Supplementary Figure 2.** Directed acyclic graph (DAG) depicting the relationships between fetal and infant weight measurements, behavior and cognitive development at 10 years and potential covariates, confounders and mediators.


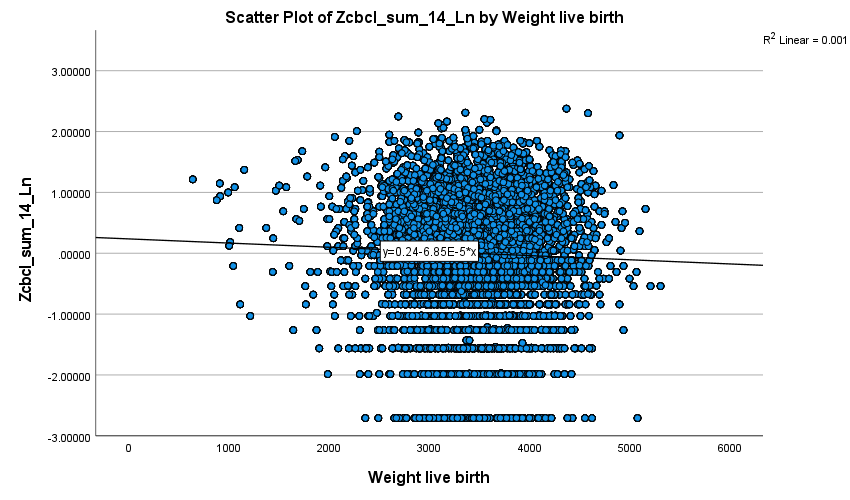

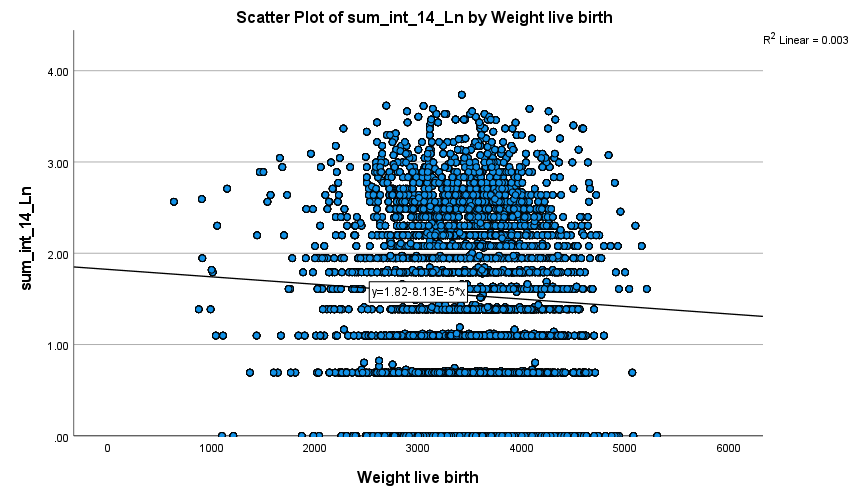

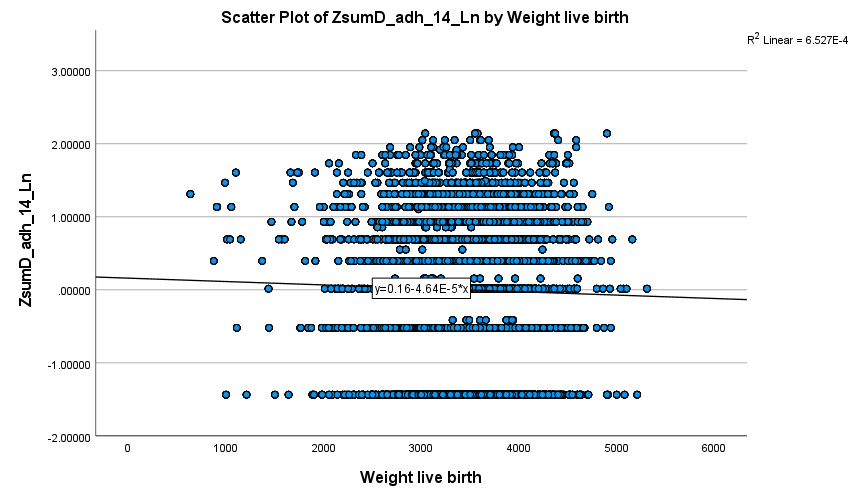


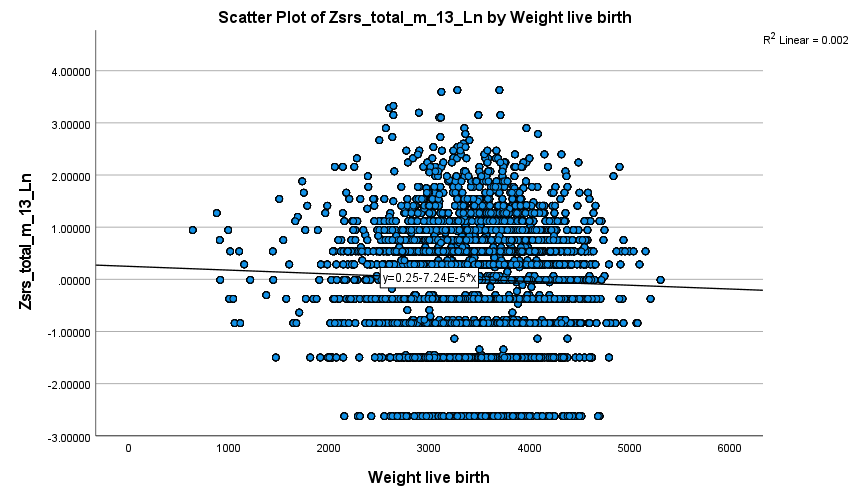

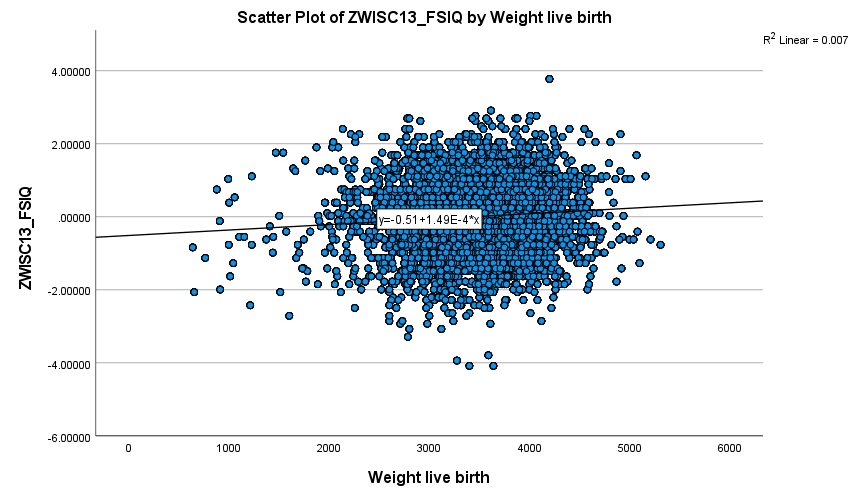

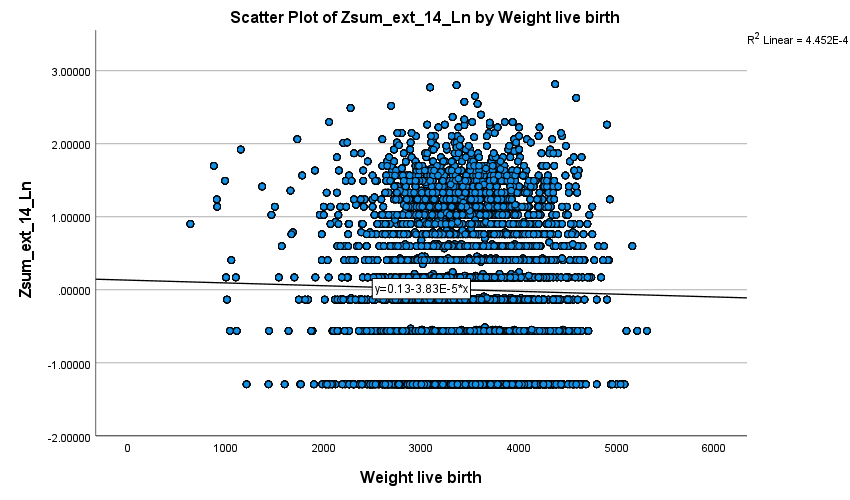


**Supplementary Figure S3. Scatterplots of birth weight with behavior and cognitive outcomes**


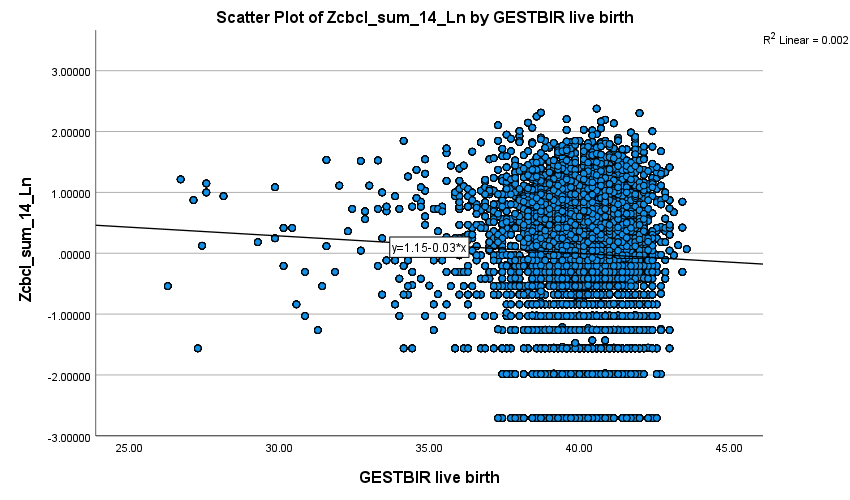

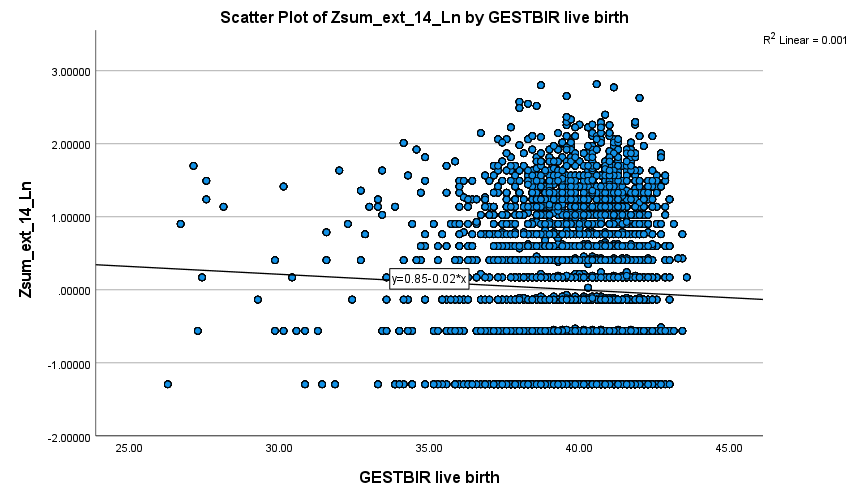

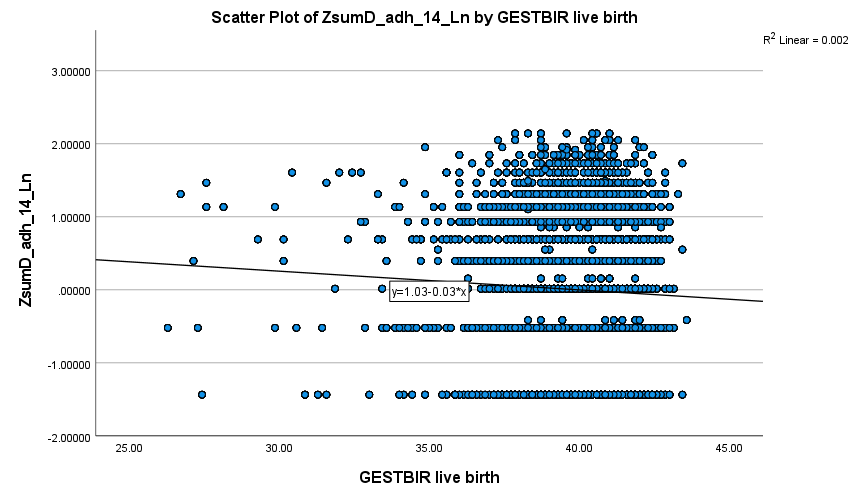


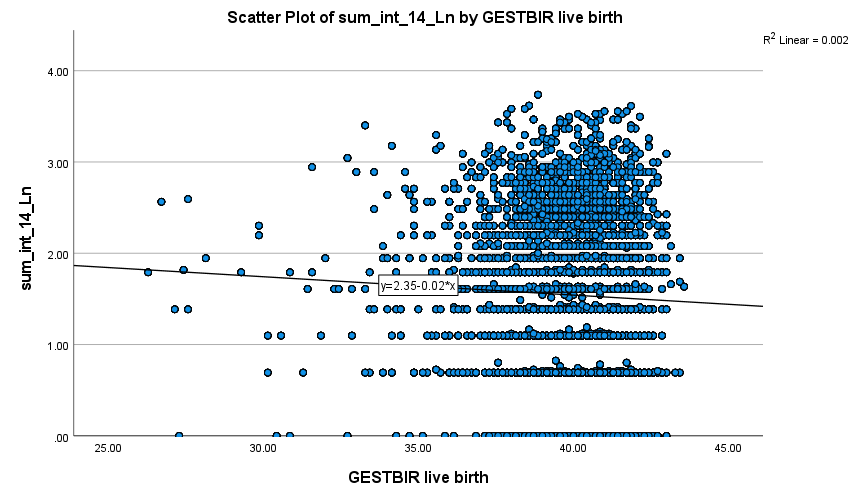

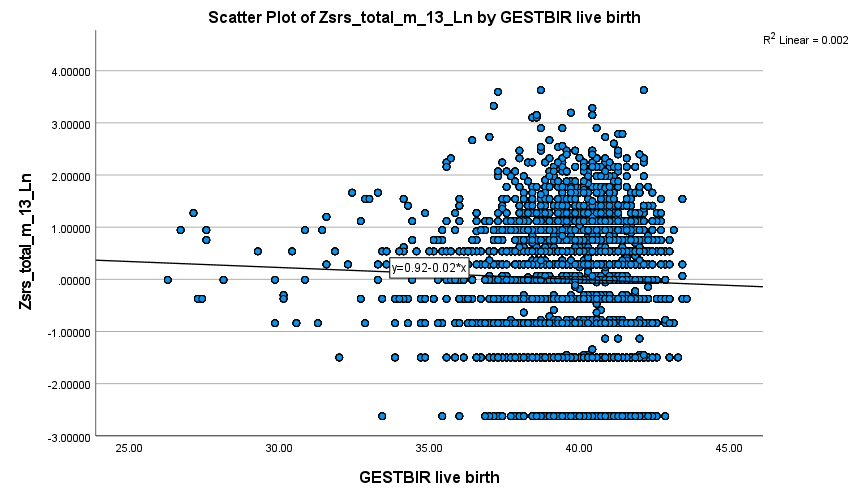

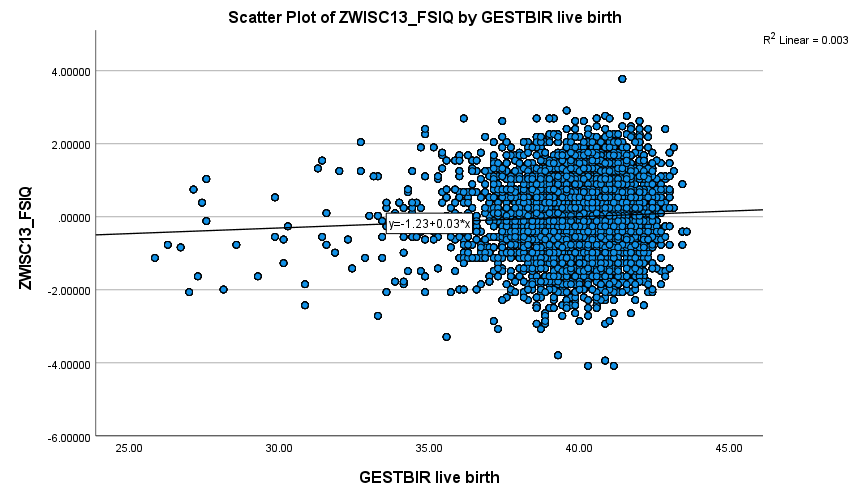


**Supplementary Figure S4. Scatterplots of gestational age at birth with behavior and cognitive outcomes**


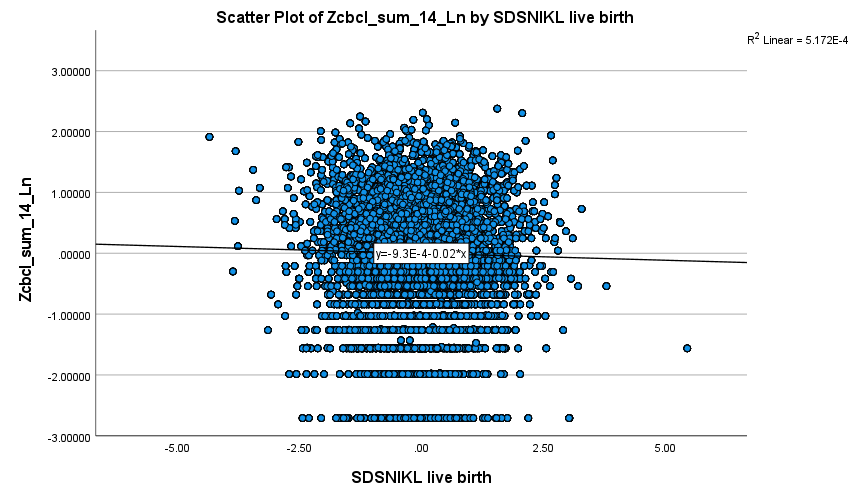

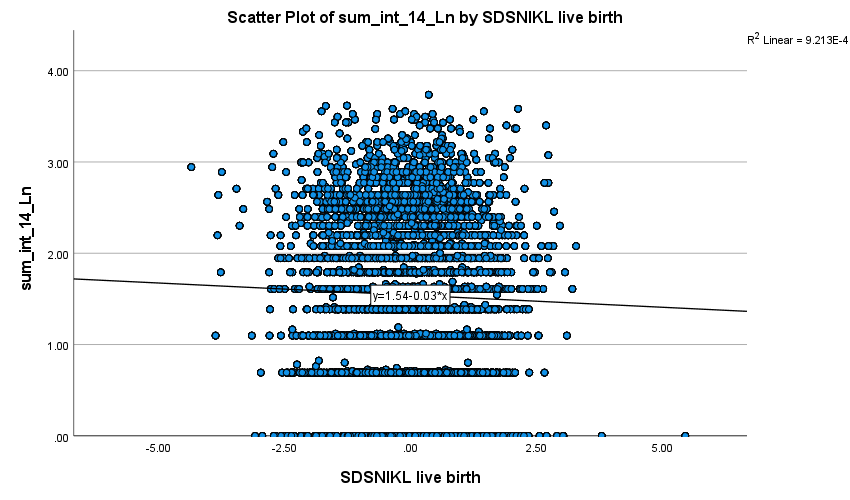

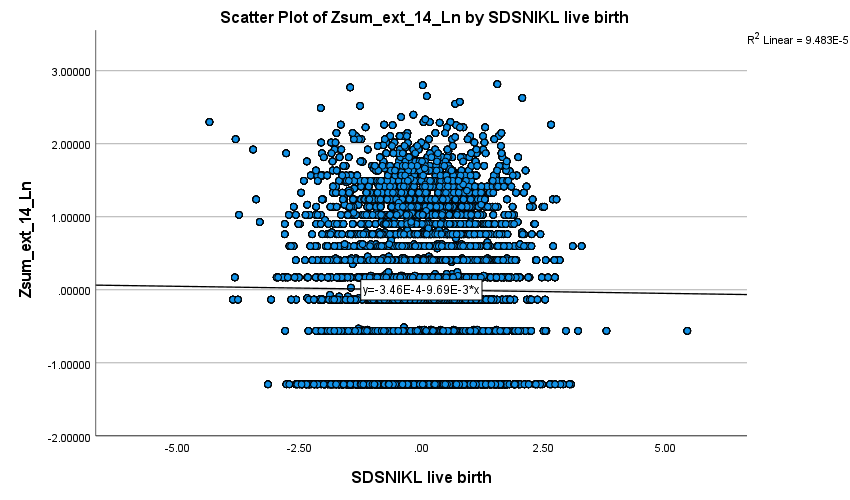

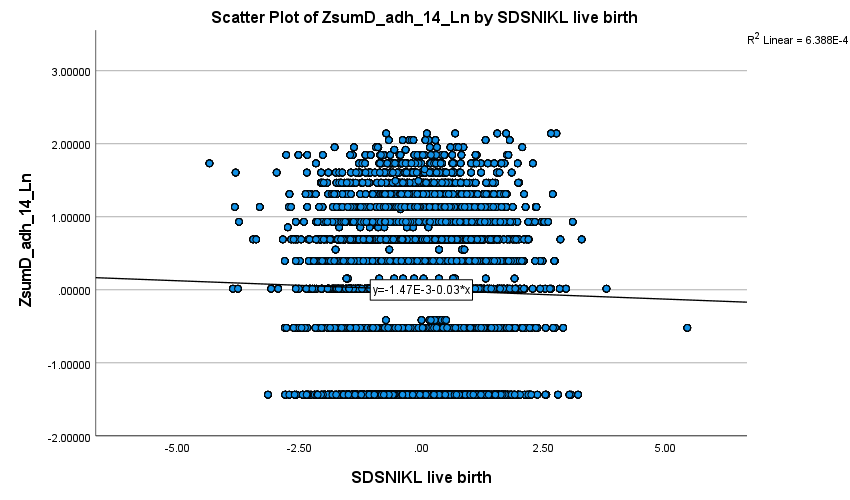

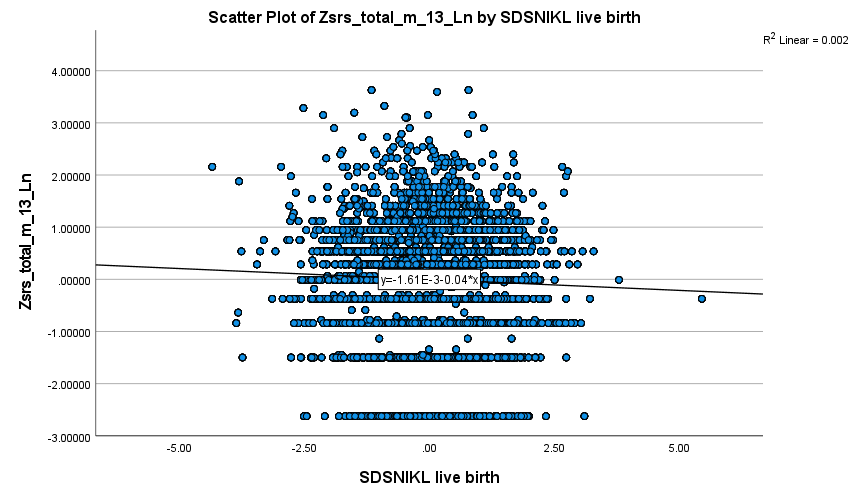


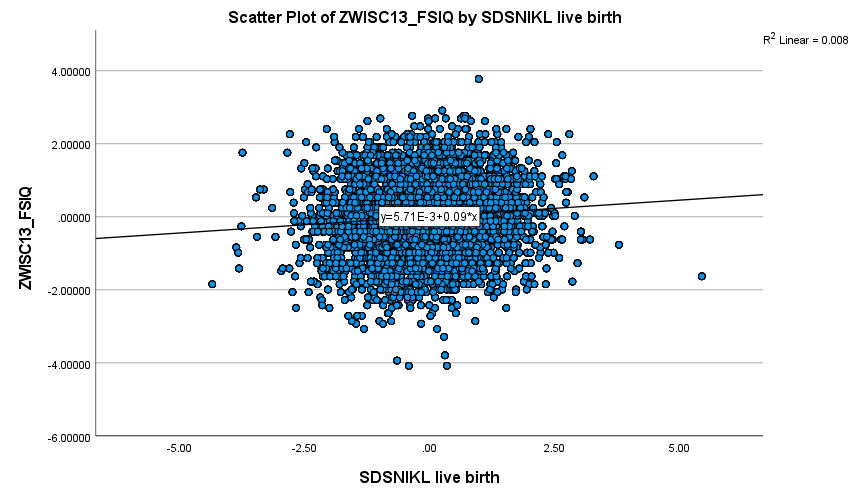


**Supplementary Figure S5. Scatterplots of gestational age and sex-adjusted birth weight with behavior and cognitive outcomes**

STROBE Statement—checklist of items that should be included in reports of observational studies

|  | | Item No. | | Recommendation | Page  No. | Relevant text from manuscript | | | | |  |
| --- | --- | --- | --- | --- | --- | --- | --- | --- | --- | --- | --- |
| **Title and abstract** | | 1 | | (*a*) Indicate the study’s design with a commonly used term in the title or the abstract | 3 | ‘Population-based prospective cohort study from fetal life until adolescence.’ | | | | |  |
|  |  |  |  | (*b*) Provide in the abstract an informative and balanced summary of what was done and what was found | 3 | Complete abstract | | | | |  |
| Introduction | | | | | | | | |  |  |  |
| Background/rationale | | 2 | | Explain the scientific background and rationale for the investigation being reported | 5 | ‘Prospective studies on the associations of different fetal and infant growth characteristics with neurocognitive and psychopathology outcomes in childhood may contribute to identification of specific critical periods and windows of opportunity in fetal and infant growth. We hypothesize that birth characteristics and different fetal and infant growth patterns are associated with behavior and cognitive outcomes in early adolescence.’ | | | | |  |
| Objectives | | 3 | | State specific objectives, including any prespecified hypotheses | 5 | ‘In this population-based prospective cohort study among 4,716 children, we examined the associations of fetal and infant weight growth patterns and birth characteristics with behavior and cognitive outcomes at the age of 13 years. Main outcomes included parent-reported scores for total behavior problems, internalizing behavior problems reflecting anxiety, depressive and somatic symptoms and externalizing behavior problems reflecting rule breaking and aggressive behavior, ADHD symptoms, ASD traits and observed IQ. We were specifically interested in identification of specific fetal and infant growth patterns.’ | | | | |  |
| Methods | | | | | | | | |  |  |  |
| Study design | | 4 | | Present key elements of study design early in the paper | 6 | Complete Methods section | | | | |  |
| Setting | | 5 | | Describe the setting, locations, and relevant dates, including periods of recruitment, exposure, follow-up, and data collection |  | ‘This study was embedded in the Generation R Study, a population-based prospective cohort study from early fetal life onwards (Kooijman et al., 2016). Pregnant women with a delivery date between April 2002 and January 2006, living in Rotterdam, the Netherlands, were eligible. Details on response and follow-up were described previously (Kooijman et al., 2016). We had information on fetal or infant growth in 8,624 singleton births. Analyses were restricted to a subgroup of (n= 4,716) children for whom we had follow-up information at 13 years (follow-up rate 54.7%). A flowchart is given in the **Supplemental Figure 1**. Written informed consent was provided by all parents and children. This study followed the Strengthening the Reporting of Observational Studies in Epidemiology (STROBE) reporting guideline (Vandenbroucke et al., 2007).’ | | | | |  |
| Participants | | 6 | | (*a*) *Cohort study*—Give the eligibility criteria, and the sources and methods of selection of participants. Describe methods of follow-up  *Case-control study*—Give the eligibility criteria, and the sources and methods of case ascertainment and control selection. Give the rationale for the choice of cases and controls  *Cross-sectional study*—Give the eligibility criteria, and the sources and methods of selection of participants | 6 | Pregnant women with a delivery date between April 2002 and January 2006, living in Rotterdam, the Netherlands, were eligible. Details on response and follow-up were described previously (Kooijman et al., 2016). | | | | |  |
|  |  |  |  | (*b*) *Cohort study*—For matched studies, give matching criteria and number of exposed and unexposed  *Case-control study*—For matched studies, give matching criteria and the number of controls per case | na | na | | | | |  |
| Variables | | 7 | | Clearly define all outcomes, exposures, predictors, potential confounders, and effect modifiers. Give diagnostic criteria, if applicable | 6-8 | Complete Methods section. | | | | |  |
| Data sources/ measurement | | 8* | | For each variable of interest, give sources of data and details of methods of assessment (measurement). Describe comparability of assessment methods if there is more than one group | 6-8 | Complete Methods section. | | | | |  |
| Bias | | 9 | | Describe any efforts to address potential sources of bias | 10 | ‘For all analyses, basic models were adjusted for child’s sex and age at outcome assessment. The confounder-adjusted model, which we considered the main model, was additionally adjusted for maternal age, parity, pre-pregnancy body mass index, educational level, ethnicity, prenatal folic acid use, smoking, alcohol use and maternal IQ at 6 years old. Potential confounders were identified based on previous literature and we selected those that fulfilled the graphical criteria for confounding in a DAG and changed the effect estimates >10% after addition to the crude model.’ | | | | |  |
| Study size | | 10 | | Explain how the study size was arrived at | 6 | ‘This study was embedded in the Generation R Study, a population-based prospective cohort study from early fetal life onwards (Kooijman et al., 2016). Pregnant women with a delivery date between April 2002 and January 2006, living in Rotterdam, the Netherlands, were eligible. Details on response and follow-up were described previously (Kooijman et al., 2016). We had information on fetal or infant growth in 8,624 singleton births. Analyses were restricted to a subgroup of (n= 4,716) children for whom we had follow-up information at 13 years (follow-up rate 54.7%). A flowchart is given in the **Supplemental Figure 1**. Written informed consent was provided by all parents and children. This study followed the Strengthening the Reporting of Observational Studies in Epidemiology (STROBE) reporting guideline (Vandenbroucke et al., 2007).’ | | | | |  |
| Quantitative variables | 11 | | Explain how quantitative variables were handled in the analyses. If applicable, describe which groupings were chosen and why | | | | 6-9 | Methods section | | |  |
| Statistical methods | 12 | | (*a*) Describe all statistical methods, including those used to control for confounding | | | | 9 | ‘ First, we described maternal, fetal and childhood characteristics. We performed a non-response analysis by comparing characteristics of children with and without outcome assessments by using Independent Student T-test, Mann-Whitney U and χ^2^ tests. Second, we used linear and logistic regression models to assess the associations of birth characteristics with continuous and dichotomous scores of behavior and cognitive outcomes at the age of 13 years. We have added scatter plots of the associations to the **Supplemental Figures S3-S5.** For all analyses, the continuous scores for total, internalizing and externalizing behavior problems, ADHD symptoms and ASD traits were natural log-transformed to deal with a skewed distribution. Third, we used estimated fetal weight growth, gestational age adjusted birth weight and infant weight and categorized both fetal weight growth (second trimester to birth) and infant weight growth (birth to 24 months) change each into three groups (growth deceleration, normal growth, and growth acceleration), and created a combined 3x3 variable leading to nine different growth patterns. We used multivariable linear or logistic regression models to explore associations of fetal and infant weight changes with continuous and dichotomous behavior and cognitive outcomes. We performed a sensitivity analysis restricting the study population to only children with weight measurements at 24 months (n=3,097). Last, to test the robustness of our findings for different growth measures, we performed a sensitivity analysis for fetal and infant head circumference. We categorized head circumference at birth (0-3 months) and during infancy (12 months) into tertiles (smallest, middle and largest) and created a combined variable that reflects 9 different head circumference patterns. Participants were included if they had head circumference data at both time points. Because of the structural difference in the fetal and childhood head circumference reference charts, we did not combine these charts. For all analyses, basic models were adjusted for child’s sex and age at outcome assessment. The confounder-adjusted model, which we considered the main model, was additionally adjusted for maternal age, parity, pre-pregnancy body mass index, educational level, ethnicity, prenatal folic acid use, smoking, alcohol use and maternal IQ at 6 years old. Potential confounders were identified based on previous literature and we selected those that fulfilled the graphical criteria for confounding in a DAG and changed the effect estimates >10% after addition to the crude model. Consistently associated with fetal growth, birth weight and infancy weight were maternal educational level, ethnicity, folic acid supplementation, smoking during pregnancy, maternal IQ and BMI at age of outcome measurement. As fetal and infant growth are highly correlated and we considered three groups of outcomes, namely CBCL derivatives, SRS scores and IQ, we took account for multiple testing by specifying significant p-values as P<(0.05/3) 0.017. We tested for statistical interaction of maternal educational level, ethnicity, smoking, alcohol use and folic acid supplementation during pregnancy and maternal IQ in these associations but no statistically significant interactions were observed (p >0.05). Missing data in covariates (ranging from 0.4 to 36.9%) were multiple imputed using the Markov Chain Monte Carlo method. Ten imputed datasets were created and analyzed together (Sterne et al., 2009). Statistical analyses were performed using the Statistical Package of Social Sciences version 25.0 for Windows (SPSS Inc., Chicago, IL, USA).’ | | |  |
|  |  |  | (*b*) Describe any methods used to examine subgroups and interactions | | | | - | Na | | |  |
|  |  |  | (*c*) Explain how missing data were addressed | | | | 9 | ‘Missing data in covariates (ranging from 0.4 to 36.9%) were multiple imputed using the Markov Chain Monte Carlo method. Ten imputed datasets were created and analyzed together (32).’ | | |  |
|  |  |  | (*d*) *Cohort study*—If applicable, explain how loss to follow-up was addressed  *Case-control study*—If applicable, explain how matching of cases and controls was addressed  *Cross-sectional study*—If applicable, describe analytical methods taking account of sampling strategy | | | | - | na | | |  |
|  |  |  | (*e*) Describe any sensitivity analyses | | | | 9 | ‘We performed a sensitivity analysis restricting the study population to only children with weight measurements at 24 months (n=3,097). Last, to test the robustness of our findings for different growth measures, we performed a sensitivity analysis for fetal and infant head circumference. We categorized head circumference at birth (0-3 months) and during infancy (12 months) into tertiles (smallest, middle and largest) and created a combined variable that reflects 9 different head circumference patterns. Participants were included if they had head circumference data at both time points. Because of the structural difference in the fetal and childhood head circumference reference charts, we did not combine these charts.’ | | |  |
| Results | | | | | | | | | | | |
| Participants | 13* | | (a) Report numbers of individuals at each stage of study—eg numbers potentially eligible, examined for eligibility, confirmed eligible, included in the study, completing follow-up, and analysed | | | | 19-27 | See table content and footnotes | | |  |
|  |  |  | (b) Give reasons for non-participation at each stage | | | | 6-9 | Methods section | | |  |
|  |  |  | (c) Consider use of a flow diagram | | | | Supplement 18 | Supplementary Figure 1 | | |  |
| Descriptive data | 14* | | (a) Give characteristics of study participants (eg demographic, clinical, social) and information on exposures and potential confounders | | | | 19, supplement 7-8 | Table 1 and Supplementary Table 1 | | |  |
|  |  |  | (b) Indicate number of participants with missing data for each variable of interest | | | | Supplement 7-8 | Supplementary Table 1 | | |  |
|  |  |  | (c) *Cohort study*—Summarise follow-up time (eg, average and total amount) | | | | 6-9 | Methods section | | |  |
| Outcome data | 15* | | *Cohort study*—Report numbers of outcome events or summary measures over time | | | | 6-9 | Methods section, Table 1 and Supplementary Table 1 | | |  |
|  |  |  | *Case-control study—*Report numbers in each exposure category, or summary measures of exposure | | | | - | na | | |  |
|  |  |  | *Cross-sectional study—*Report numbers of outcome events or summary measures | | | | - | Na | | |  |
| Main results | 16 | | (*a*) Give unadjusted estimates and, if applicable, confounder-adjusted estimates and their precision (eg, 95% confidence interval). Make clear which confounders were adjusted for and why they were included | | | | 9-11, 19-27 | Statistical analysis section of Methods. Results section. Tables and footnotes. | | |  |
|  |  |  | (*b*) Report category boundaries when continuous variables were categorized | | | | 6-9 | Methods section | | |  |
|  |  |  | (*c*) If relevant, consider translating estimates of relative risk into absolute risk for a meaningful time period | | | | - | Na. | | |  |

Continued on next page

| Other analyses | 17 | Report other analyses done—eg analyses of subgroups and interactions, and sensitivity analyses | 9 | ‘We performed a sensitivity analysis restricting the study population to only children with weight measurements at 24 months (n=3,097). Last, to test the robustness of our findings for different growth measures, we performed a sensitivity analysis for fetal and infant head circumference. We categorized head circumference at birth (0-3 months) and during infancy (12 months) into tertiles (smallest, middle and largest) and created a combined variable that reflects 9 different head circumference patterns. Participants were included if they had head circumference data at both time points. Because of the structural difference in the fetal and childhood head circumference reference charts, we did not combine these charts.’ |  |
| --- | --- | --- | --- | --- | --- |
| Discussion | | | | | |
| Key results | 18 | Summarise key results with reference to study objectives | 12 | ‘In this population-based prospective cohort study, we observed that a longer gestational age at birth was associated with lower parent-reported scores for total behavior problems and ADHD symptoms in early adolescence. Increased birth weight was associated with lower odds of parent-reported high externalizing problems and ADHD symptoms, and lower odds of a low IQ. Children born SGA had higher parent-reported total and internalizing behavior problems, ADHD symptoms, and lower IQ in early adolescence, compared to those born AGA. Children with accelerated fetal and infant growth had a higher IQ in early adolescence, whereas those with decelerated fetal growth followed by normal infant growth had lower odds of a high parent-reported total behavior problems score. The associations of fetal and infant head circumference with behavior and cognitive outcomes were stronger than those of fetal and infant weight growth patterns with the same behavioral and cognitive outcomes.’ |  |
| Limitations | 19 | Discuss limitations of the study, taking into account sources of potential bias or imprecision. Discuss both direction and magnitude of any potential bias | 14 | ‘Strengths of this study include the study design, large number of participants, detailed data on weight measurements from second trimester up to two years of age and extensive report on behavioral and cognitive outcomes. In addition to previous research conducted in our study population examining similar associations, this study is of added value since it is conducted in older children and adds fetal-infant growth patterns. This study also has limitations. Of the 8,624 singleton live births with information on fetal or infant growth, 4,716 children had data regarding behavior and cognitive outcome measurements. Mothers of children not included in our analyses were younger, less often primipara, higher educated and of European ethnicity, had lower IQ and less often used alcohol during pregnancy. This seems to suggest bias to a relatively more healthy population and might affect the generalizability of our results. Previous research has shown that the association of birth weight and gestational age at birth with IQ or behavior in later life might not be linear. The positive effect of gestational age on IQ seems greater below 34 weeks of gestation as compared to 34-37 weeks of gestation (Eves et al., 2023; MacKay et al., 2010; Wolke et al., 2015). In our population the number of preterm born children is small (4.6%) and an even smaller number of those born preterm was below <34 weeks (1.1%). Therefore we did not have enough numbers to stratify on gestational age or even on term birth versus preterm birth. Such stratification might be relevant for studies with larger numbers since rapid infant weight gain may be stronger associated with neurodevelopmental benefits for preterm infants than term infants (Belfort et al., 2010). Furthermore, the measurement of estimated fetal weight can be inaccurate and random error might be high, especially in the extreme values of estimated fetal weight (Dudley, 2005; Ewington et al., 2024). The stronger effects of postnatal weight growth than fetal weight growth might be due to biological effects or the results of better precision for postnatal measurements. We were interested in the combination between fetal and infant growth delta’s, which are in line with clinical guidelines regarding growth curves and the cut-offs used for growth deceleration and acceleration respectively. Further studies might focused on more detailed growth modelling aiming to identify the optimal growth patterns in early life for mental health outcomes. Although we adjusted for a large number of potential confounders, residual confounding might still be a possibility due to the observational nature of the study.’ |  |
| Interpretation | 20 | Give a cautious overall interpretation of results considering objectives, limitations, multiplicity of analyses, results from similar studies, and other relevant evidence | 12-14 | Discussion |  |
| Generalisability | 21 | Discuss the generalisability (external validity) of the study results | 14 | ‘Mothers of children not included in our analyses were younger, less often primipara, higher educated and of European ethnicity, had lower IQ and less often used alcohol during pregnancy. This seems to suggest bias to a relatively more healthy population and might affect the generalizability of our results.’ |  |
| Other information | |  | | | |
| Funding | 22 | Give the source of funding and the role of the funders for the present study and, if applicable, for the original study on which the present article is based | 15 | ‘ This work was supported by the European Research Council [Consolidator Grant, ERC-2014-CoG-648916, received by Prof. Vincent V.W. Jaddoe], the European Joint Programming Initiative “A Healthy Diet for a Healthy Life” (JPI HDHL) the Netherlands, [EndObesity, ZonMW grant number 529051026, received by Dr Romy Gaillard], the European Union's Horizon 2020 Research and Innovation Program grant agreement, 733206 (LifeCycle) and 874583 (ATHLETE Project). The Netherlands Organization for Health Research and Development [NWO, ZonMW, grant number 543003109, received by Dr Romy Gaillard], and the LEaDing Fellows EU Marie Skłodowska-Curie COFUND Programme grant 707404 (Dr Sammallahti). Hanan El Marroun was supported by Stichting Volksbond Rotterdam, the Brain & Behavior Research Foundation (NARSAD Young Investigator Grant 27853), the Netherlands Organization for Health Research and Development (Aspasia grant No. 015.016.056). Support for Kelly K. Ferguson was provided by the Intramural Research Program of the National Institute of Environmental Health Sciences, National Institutes of Health. The Generation R Study is financially supported by the Erasmus Medical Center, Rotterdam, the Erasmus University Rotterdam and the Netherlands Organization for Health Research and Development and the Ministry of Health, Welfare and Sport.’ |  |

*Give information separately for cases and controls in case-control studies and, if applicable, for exposed and unexposed groups in cohort and cross-sectional studies.

**Note:** An Explanation and Elaboration article discusses each checklist item and gives methodological background and published examples of transparent reporting. The STROBE checklist is best used in conjunction with this article (freely available on the Web sites of PLoS Medicine at http://www.plosmedicine.org/, Annals of Internal Medicine at http://www.annals.org/, and Epidemiology at http://www.epidem.com/). Information on the STROBE Initiative is available at www.strobe-statement.org.
